# Biocompatible copper formate-based nanoparticles with strong antibacterial properties for wound healing

**DOI:** 10.1186/s12951-023-02247-w

**Published:** 2023-12-10

**Authors:** Yue Zhou, Ping Sun, Yongbin Cao, Jiahao Yang, Qingzhi Wu, Jian Peng

**Affiliations:** 1 State Key Laboratory of Advanced Technology for Materials Synthesis and Processing, Wuhan, 430070 China; 2 School of Chemistry, Chemical Engineering and Life Science, Wuhan University of Technology, Wuhan, 430070 China; 3 School of Material Science and Engineering, Wuhan University of Technology, Wuhan, 430070 China

**Keywords:** Cuf-TMB@PDA, Bioactive, Antibacterial, ·OH radical, Wound healing

## Abstract

Copper-based antibacterial materials have emerged as a potential alternative for combating bacterial infections, which continue to pose significant health risks. Nevertheless, the use of copper-based nanoparticles as antibacterial agents has faced challenges due to their toxicity towards cells and tissues. To overcome this obstacle, we propose a new approach using a contact-active copper-based nanoparticles called polydopamine (PDA)-coated copper-amine (Cuf-TMB@PDA). The positively charged surface of Cuf-TMB@PDA enables efficient targeting of negatively charged bacteria, allowing controlled release of Cu(II) into the bacterial cell membrane. Moreover, Cuf-TMB@PDA exhibits similar ·OH signals as Cuf-TMB suspensions in previous work. In cytotoxicity assays conducted over 72 h, Cuf-TMB@PDA demonstrated an efficacy of 98.56%, while releasing lower levels of Cu(II) that were less harmful to cells, resulting in enhanced antimicrobial effects. These antimicrobial properties are attributed to the synergistic effects of charge-contact activity of PDA, controlled release of Cu(II), and free radicals. Subsequent in vivo experiments confirmed the strong antimicrobial potency of Cuf-TMB@PDA and its ability to promote wound healing.

**Supplementary Information:**

The online version contains supplementary material available at 10.1186/s12951-023-02247-w.

## Introduction

Microbiologically induced diseases which could disseminated through the food chain, pose substantial hazards and financial burdens to human society [1–3]. Pathogenic microorganisms like Escherichia coli (*E. coli*), Staphylococcus aureus (*S. aureus*), and others have been linked to various infections, including wound infections, bloodstream infections, and systemic infections [4–10]. The rise of antibiotic-resistant bacteria rendered conventional antibiotic treatments are no longer effective at completely eradicating these illnesses [11–13]. Therefore, eliminating superbugs and addressing bacterial drug resistance have become crucial research areas in the biomedical field. Historically, metals such as gold [14], silver [15, 16], copper [17, 18], and zinc [19] have been utilized as antibacterial agents in various fields, including medicine and environmental studies [20–23]. Among them, copper possesses antimicrobial properties comparable to those of silver [24, 25], and serves as an essential co-factor for organisms, promoting vital processes such as vascular growth, collagen deposition, and wound re-epithelialization [26, 27]. Nevertheless, copper can be detrimental when its amount exceeds the threshold established by longstanding evolutionary homeostatic mechanisms.

Considerable research efforts have been focused on strategies aimed at reducing the utilization of copper or mitigating its release rate [24, 28–30]. For instance, Ameer and colleagues utilized folic acid as a stabilizing agent for copper metal-organic frameworks (Cu-MOFs), resulting in a decrease in their degradation rate and the release rate of Cu(II) ions. This approach effectively alleviated the harmful toxic effects associated with Cu(II) ions [25]. Du et al. proposed a methodology for incorporating silver onto Poly-Cu MOFs, resulting in a lower copper node density and a higher loading of Ag NPs. This approach led to a reduced release of Cu(II) ions and an enhanced release of Ag(I) ions, ultimately improving biocompatibility and heightening antimicrobial properties [28]. Furthermore, various biocompatible nanomaterials, including polydopamine [31, 32], chitosan [33], carbon quantum dots [34], tannic acid [35] and their derivatives [36, 37], have been developed as effective ligands for copper complexes [38–40]. The coordination of copper with these ligands not only results in a reduction in copper content, but also allows for synergistically harnessing of their properties, achieving a more potent sterilization effect and enhanced biocompatibility. Booccaccini et al. formulated a biodegradable zein/CuBG that releases Cu(II) under physiological conditions, inducing oxidative stress in bacteria and effectively inhibiting their growth [40]. Zhang et al. introduced Cu_x_O-PDA, a composite that instigated DNA degradation, lipid peroxidation, and the eradication of biofilms. This composite demonstrated effective bacteriostatic properties against both *E. coli* and *S.aureus* [41]. Despite the abundance of reports on copper-based antimicrobial materials, the development of copper-based materials that possess both cytocompatibility and strong antimicrobial properties have been relatively limited until now.

Herein, polydopamine-coated Cuf-TMB (Cuf-TMB@PDA) nanoparticles were designed and utilized to investigate their antimicrobial properties against two representative bacterial strains for wound healing. Catechol groups present on polydopamine demonstrate superior adhesion and compatibility with cells and tissues. Additionally, this positively charged PDA layer can adsorb onto the negatively charged bacterial cell membrane surface through electrostatic interactions, thus impeding bacterial nutrient uptake. Furthermore, it was demonstrated that Cuf-TMB@PDA exhibited signals of hydroxyl radicals (·OH) in its electron spin resonance (ESR) spectrum. Due to the significant aggregation of Cuf-TMB@PDA on bacterial surfaces, gradual release of Cu(II), and damage caused by ·OH within a specific range, the antibacterial efficacy of Cuf-TMB@PDA is significantly enhanced. Importantly, the obtained Cuf-TMB@PDA not only exhibited effective antibacterial activity in vitro experiments but also demonstrated good biocompatibility. Subsequent in vivo experiments further confirmed the enhanced antibacterial effect of Cuf-TMB@PDA against *S. aureus*, as well as its more pronounced effect on skin wound healing.

## Experimental section

### Materials

Copper formate tetrahydrate (Cuf) and dopamine were purchased from Macklin Biochemical Co., Ltd (Shanghai, China). 3,3’,5,5’-Tetramethylbenzidine and sodium chloride were obtained from J&K Scientific (Beijing, China). Escherichia coli (*E. coli*) and Staphylococcus aureus (*S. aureus*) were purchased from Shanghai Luwei Technology Co., Ltd (Shanghai, China). Mice dermal fibroblasts were obtained from Bena Culture Collection (Beijing, China). L929 cells were cultured in Dulbecco’s modified Eagle’s medium (DMEM, glucose concentration: 4.5 g L^− 1^) containing 10% FBS, 100 unit mL^− 1^ penicillin G sodium, and 100 µg mL^− 1^ streptomycin sulfate. All cells were maintained in an incubator at 37 °C in a humidified environment with 5% CO_2_ in the air. Cell counting kit-8 (CCK-8) was obtained by the biosharp Co., Ltd (Guangzhou, China). Healthy Mice (Bablc/6) were obtained from Hubei Laboratory Animal Research Center (Wuhan, China). 5,5-dimethyl-1-pyrroline-n-oxide (DMPO) was purchased from Dojindo Molecular Technology Co., Ltd (Tokyo, Japan). The ultrapure water used in all experiments was made by passing through an ultrapure purification system (Ulupure, UPH-l-10TN). The animal protocols were approved by the Institutional Animal Care and Use Committee (IACUC) of Wuhan University of Technology.

### Synthesis of the Cuf-TMB@PDA

The preparation of the Cuf-TMB nanoparticles was followed to our previous work [42]. For the synthesis of Cuf-TMB@PDA, dopamine (DA) solution (0.1 µg mL^− 1^) was mixed with Cuf-TMB suspension (78 µg mL^− 1^) in equal volumes under stirring at 380 rpm for 80 min at room temperature. This suspension was subsequently dialyzed for 48 h to remove any unreacted reagent and ions. The Cuf-TMB@PDA was freeze-dried and stored at 4 ^o^C until further use.

### Characterization

The crystal structure of the Cuf-TMB@PDA samples was examined by X-ray diffraction (XRD, Rigaku Smart Lab). The morphological characterization of the prepared samples was obtained by scanning electron microscopy (SEM, HITACHI SU8010), transmission electron microscopy (TEM, JEM-1400), and high-resolution TEM (HRTEM, Thermo Scientific Talos F200X G2). The molecular structure and bond information of Cuf-TMB@PDA were obtained with Fourier transform infrared spectroscopy (FT-IR, Nicolet 6700) and laser confocal micro-Raman spectroscopy (Xplora PLUS). The composition and chemical state of the elements were characterized using X-ray photoelectron spectroscopy (XPS, Thermo Fisher Scientific K-Alpha+). The peroxidase-like (POD) activity of Cuf-TMB NPs was tested by UV-1900i Visible-near-infrared spectrophotometer (Shimadzu, Japan).

### In vitro antibacterial property of Cuf-TMB@PDA

*E. coli* and *S. aureus* were chosen as the representative to evaluate the antibacterial activity of Cuf-TMB@PDA. These bacteria were incubated in liquid Luria-Bertani (LB) culture medium by shaking at 37 °C for 18 h. The optical density (OD) values of *E. coli* or *S. aureus* suspension were optimized at 0.3. Then, *E. coli* or *S. aureus* suspensions (500 µL) were incubated with Cuf-TMB@PDA, Cuf-TMB, and PBS (control group) at 37 °C, respectively. After 24 h, the live/dead staining cells of bacterial cells was determined using the microscope. The morphology was observed using SEM.

### In vitro biocompatibility evaluation of Cuf-TMB@PDA

The cytocompatibility of Cuf-TMB@PDA was evaluated by the standard CCK-8 method. L929 cells were cultured for a further 24 h with various Cuf-TMB@PDA concentrations (0.001˗63 µg mL^− 1^). 100 µL of fresh cell culture medium and 10 µL of CCK-8 solution were successively added to each well after washing with PBS. A microplate reader was used to measure the absorbance of each well at 495 nm after 4-hour incubation. The cell viabilities were expressed by the following equation:

1 $$Cell \, Viability \left(\%\right)=\left(\raisebox{1ex}{${A}_{1}$}\!\left/ \!\raisebox{-1ex}{${A}_{0}$}\right.\right)\times 100\%$$

Where A_1_ and A_0_ represented the absorbance value of the experimental and control groups.

Live/dead cell staining assays, cells were exposed to medium-diluted Cuf-TMB@PDA for 24 h. Cells without Cuf-TMB@PDA treatment served as the control group. Subsequently, live/dead staining was applied to the cell mixture, followed by an incubation period. The stained L929 fibroblasts were subsequently examined using an inverted fluorescence microscope.

### In vivo wound closure evaluation of the Cuf-TMB@PDA

The wound-healing performance of Cuf-TMB@PDA was evaluated utilizing a full-thickness rat skin incision model with certain modifications from previously reported methods [41]. All animal experiments received approval from the Institutional Review Board of Wuhan University of Technology. The infected wounds were divided into three groups: PBS, Cuf-TMB, and Cuf-TMB@PDA. At specific intervals, namely, on days 3, 5, and 7 post-treatment, the wound size and body weight of the mice were recorded.

The wound closer ratio was calculated by the following formula:

2 $$Wound \, concentration \, area \left(\%\right)= \frac{{Area}_{ 0 \,day}- {Area}_{ n \, day}}{{Area}_{ 0 \, day}} \times 100\%$$

### In vivo histological analysis and toxicity assessment

At the completion of the wound healing experiment, the infected wound tissues and major organs of the mice were fixed in a 10% formalin solution for 24 h. Following ethanol dehydration, the tissues underwent paraffin embedding and were then sliced into 5 μm sections, subsequently stained with hematoxylin and eosin (H&E). The organ slices were observed using an Olympus FV1200 microscope. Blood samples were collected from the different treatment mice post in vivo wound healing experiment for subsequent biochemical analysis.

## Results and discussions

### Preparation and characterization of the Cuf-TMB@PDA

**Fig. 1:**
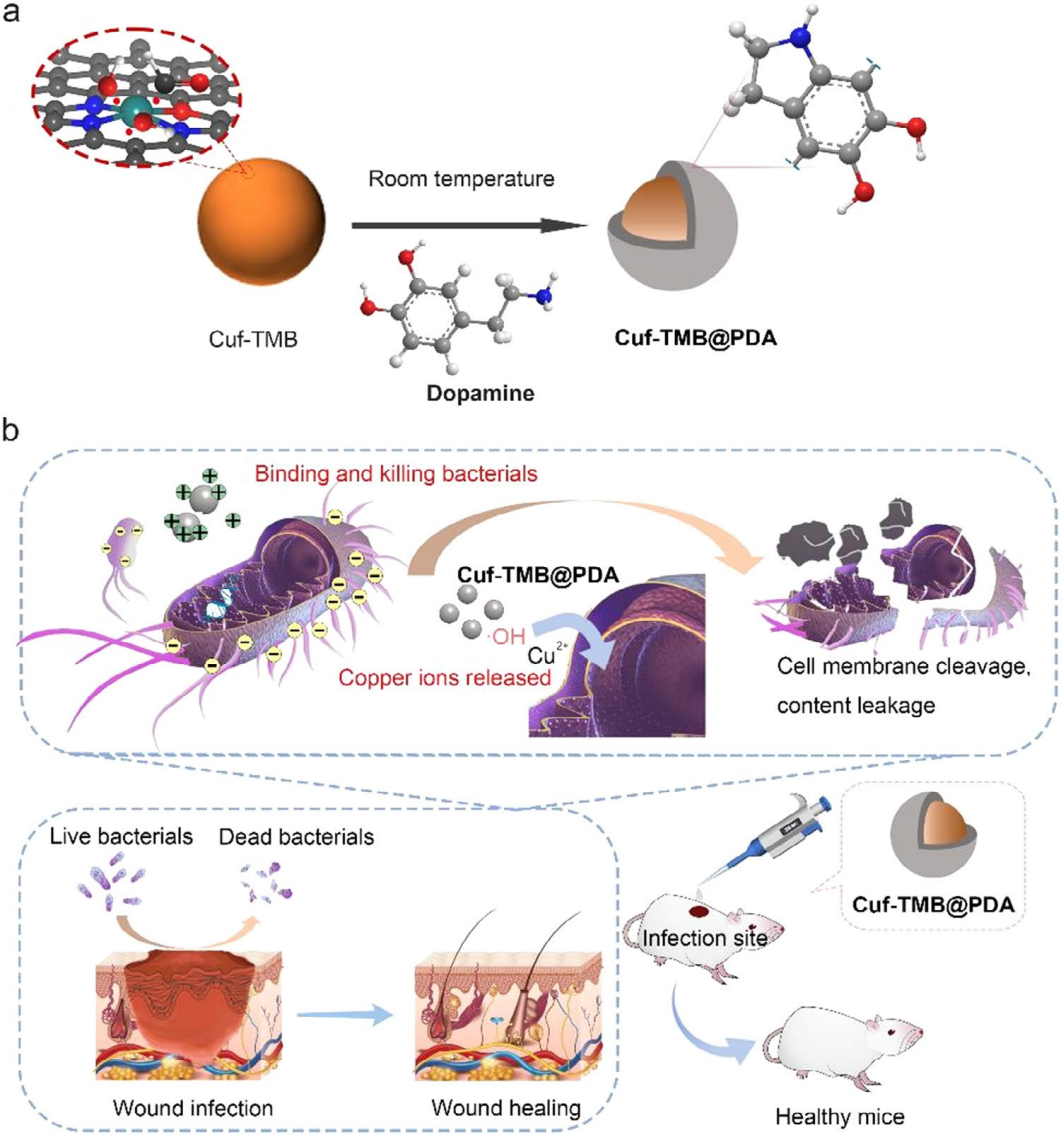
Schematic illustration of the design and synthesis of the antimicrobial Cuf-TMB@PDA for promoting skin wound healing. (**a**) The synthetic pathway of Cuf-TMB@PDA; (**b**) Schematic representation depicting the antimicrobial attributes and wound-healing promotion of Cuf-TMB@PDA. The Cuf-TMB@PDA composite demonstrates potent antimicrobial characteristics well-suited for enduring in vivo antimicrobial interventions and improved wound healing

**Fig. 2:**
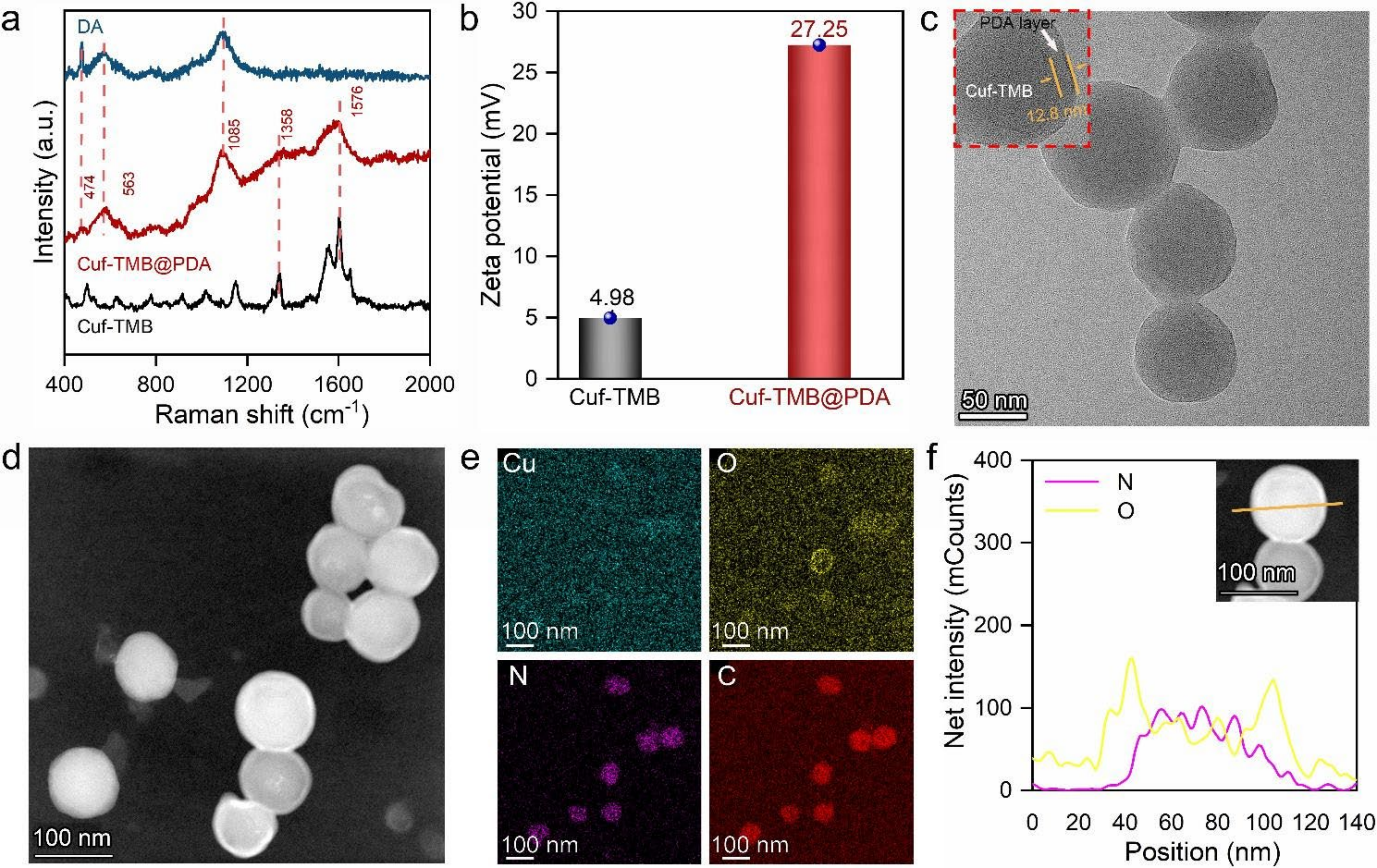
Characterization of Cuf-TMB@PDA. (**a**) Raman spectra of the Cuf-TMB@PDA, Cuf-TMB, and dopamine. (**b**) Zeta potentials of the Cuf-TMB@PDA and Cuf-TMB. (**c**) Transmission electron microscope (TEM) image of Cuf-TMB@PDA (scale bar, 50 nm). (**d**) High angle annular dark field scanning transmission electron microscopy (HAADF-STEM) image. (**e**) The corresponding energy dispersive spectrometer (EDS) mapping images of Cuf-TMB@PDA (scale bar, 100 nm). (**f**) Representative EDX line scans for N and O, obtained on Cuf-TMB@PDA (orange line, scanned line)

The typical synthetic procedure of the Cuf-TMB@PDA is schematically elucidated in Fig. 1. In this study, dopamine polymerization can be triggered by the presence of ·OH and ·CHO within the Cuf-TMB system (Scheme S1). Polydopamine (PDA) and Cuf-TMB were employed to fabricate a biocompatible and effective nanoparticle, as confirmed by the Raman spectra (Fig. 2a) and Transform Infrared spectroscopy (FT-IR) spectra (Fig. S1, Table S1). The Raman characteristic peaks at 474, 563 and 1085 cm^− 1^ were assigned to trimethylammonium groups, −NH, and − CH_2_ groups [43, 44], respectively. The zeta potential of Cuf-TMB was shifted from 4.98 to 27.25 mV after PDA coating (Fig. 2b, Fig. S2), indicating the successful formation of Cuf-TMB@PDA.

Furthermore, the XRD pattern of the synthesized Cuf-TMB@PDA exhibited an amorphous nature, consistent with the amorphous state observed in both Cuf-TMB and PDA (Fig. S3). High-angle annular dark-field scanning transmission electron microscopy (HAADF-STEM) images depicted an amorphous structure, with no noticeable boundary between the core and shell (Fig. S4). Simultaneously, a uniform and tightly adhered polydopamine (PDA) layer, approximately 10 nm thick, was consistently observed, intricately covering the spherical surface of Cuf-TMB. This observation indicates a robust binding between PDA and Cuf-TMB. Furthermore, the sizes of both Cuf-TMB and Cuf-TMB@PDA, as determined through TEM images, were in good accordance with the size distribution assay (Fig. 2c and d, and Fig. S5). The energy-dispersive X-ray (EDX) mapping showed that Cu element was concentrated in the center of the nanoparticle, while O, N and C elements dispersed across the image area. An interesting observation was the distinct oxygen distribution pattern compared to Cuf-TMB, where oxygen was primarily located at the particle edges (Fig. 2e and f, and Fig. S6). This discrepancy can be attributed to the oxidation of dopamine into polydopamine (PDA) catalyzed by radicals from Cuf-TMB suspensions. Elemental analysis (Table S2) further provides conclusive evidence for the successful synthesis of Cuf-TMB@PDA.

**Fig. 3:**
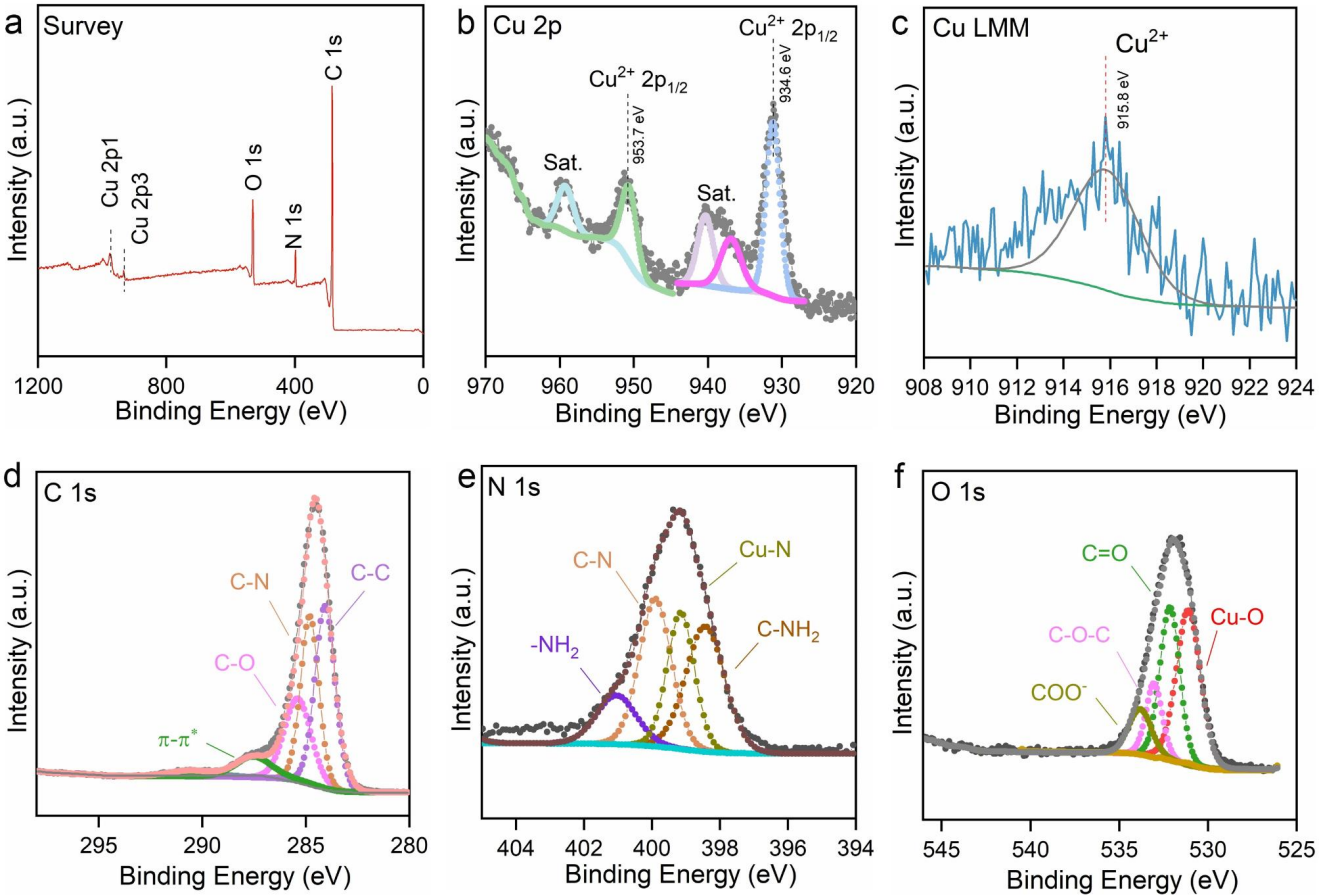
The XPS spectra of Cuf-TMB@PDA. (**a**) Fully scanned XPS spectrum of Cuf-TMB@PDA; (**b**) Spectrum depicting the Cu 2p region, featuring two primary peaks and three satellite peaks; (**c**) Cu Auger spectrum of Cuf-TMB@PDA; (**d**) The C 1s spectrum of Cuf-TMB@PDA; (**e**) The N 1s spectrum; (**f**) The O 1s spectrum

The surface composition and valence states of each element in Cuf-TMB@PDA were characterized using X-ray photoelectron spectroscopy (XPS) technique (Fig. 3). Analysis of the survey spectrum indicated the presence of predominant elements including copper (Cu), nitrogen (N), carbon (C), and oxygen (O) in Cuf-TMB@PDA (Fig. 3a). Decomposition of the Cu 2p XPS spectrum revealed distinctive features, including a Cu 2p1/2 peak at 953.7 eV, a Cu 2p3/2 peak at 934.6 eV, and three supplementary satellite peaks located at 960.3, 940.5, and 937.6 eV (Fig. 3b). These supplementary peaks typically arise from an “oscillatory” mechanism where excess electrons transition to higher energy states [45]. The predominance of Cu(II) as the primary chemical state of copper on the surface of Cuf-TMB@PDA is further supported by the Auger spectrum (Fig. 3c). The C 1s spectrum exhibits peaks at 291.0, 287.6, 285.13, and 284.8 eV, corresponding to the presence of π-π*, C–O, C–N, and C–C groups, respectively (Fig. 3d). The presence of the N 1s peak confirmed the successful formation of the PDA shell layer, as indicated by peaks at approximately 398.4 eV, representing the presence of C-NH_2_ groups. Furthermore, the N 1s XPS spectrum revealed the coexistence of Cu-N (399.5 eV), C-N (399.9 eV), and -NH_2_ (401.0 eV) species (Fig. 3e). The situation with oxygen was distinct, as the local environment of oxygen experienced changes following PDA modification, which were detectable. The O 1s spectrum of Cuf-TMB@PDA showed the presence of COO- (533.8 eV), C = O (532.1 eV), and Cu-O (531.1 eV) groups (Fig. 3f). Notably, the unique structure of PDA likely contributed to the elevated signal energy observed in the C-O-C group (533.0 eV) of Cuf-TMB@PDA, in contrast to the C-O-C moiety identified in Cuf-TMB (Fig. 3f, Fig. S7). These experimental findings not only verified the successful synthesis of Cuf-TMB@PDA but also provided evidence for the connection between PDA and Cuf-TMB through a robust C-O-C bond and C-NH_2_ structure [46, 47].

Additionally, Cuf-TMB@PDA demonstrates a specific surface area of 101.01 cm^2^ g^− 1^, contrasting with the 44.13 cm^2^ g^− 1^ observed for Cuf-TMB alone. The incorporation of PDA as an encapsulating layer transforms Cuf-TMB@PDA into a core-shell structure, thereby enhancing its specific surface area Fig. S8). Notably, the POD-like activity of Cuf-TMB@PDA remains relatively stable even when exposed to a concentration of 0.1 µg mL^− 1^ dopamine (Fig. S9, Fig. S10). Optimal performance of Cuf-TMB@PDA is achieved under conditions of 40 ℃ and at a specific pH level of 7 (Fig. S11).

### The vitro cell compatibility and hemocompatibility property of Cuf-TMB@PDA

**Fig. 4:**
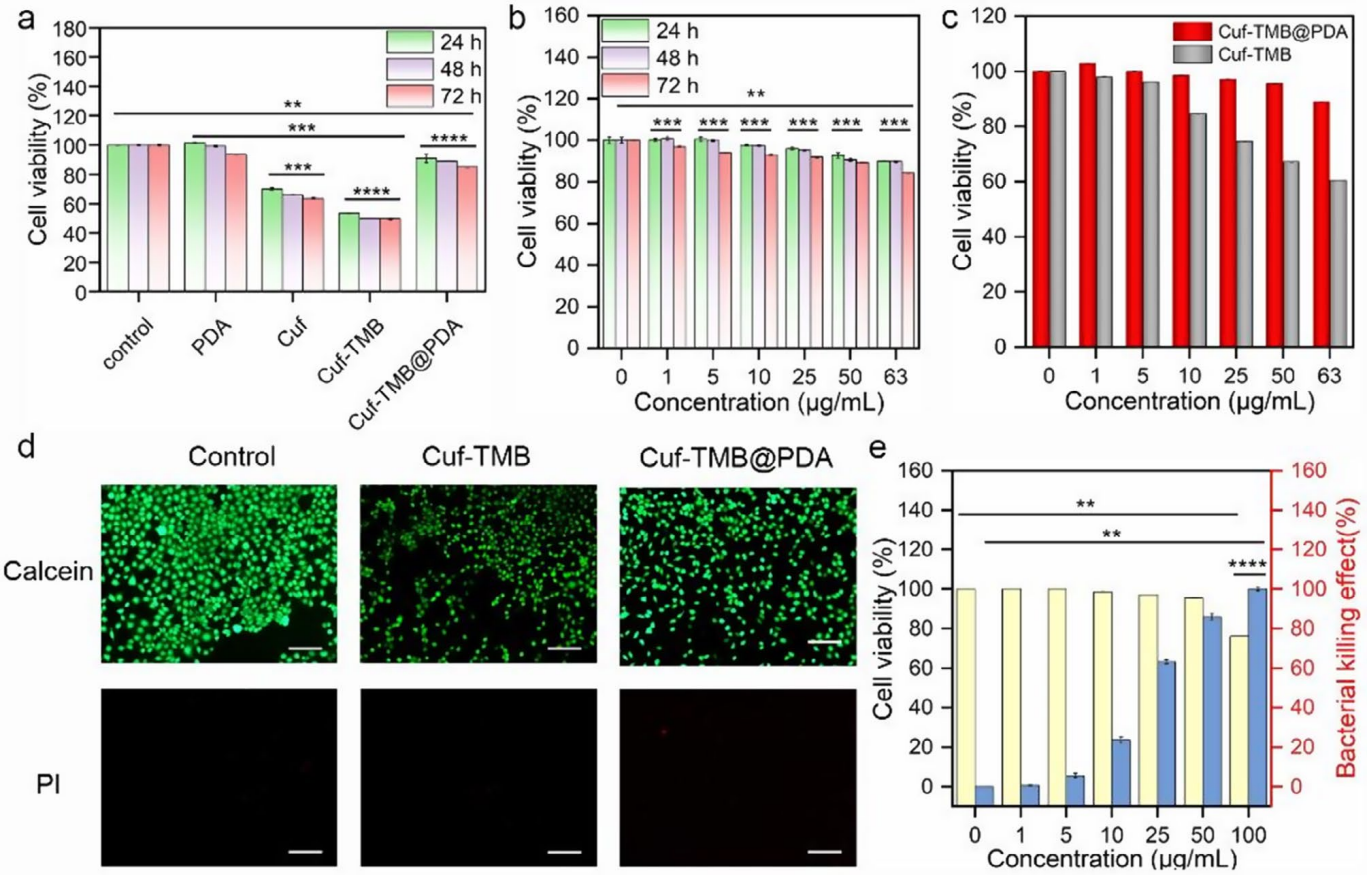
Evaluation of the cytocompatibility of Cuf-TMB@PDA. (**a**) The viability of L929 cells was examined after exposure to various treatments for durations of 24, 48, and 72 h, respectively. (**b**) Viability of L929 cells after incubation in leaching solutions with different concentrations for 24 h, 48 h, and 72 h. (**c**) Viability of L929 cells after incubation in leaching solutions with varying concentrations of Cuf-TMB@PDA and Cuf-TMB for 24 h. (**d**) Live/dead staining results of L929 cells following incubation in the leaching solution of Cuf-TMB@PDA (63 µg mL^− 1^) for 24 h (scale bar: 200 μm). (**e**) Determination of the optimal Cuf-TMB@PDA concentration for both cell cytocompatibility and antibacterial activity (*P < 0.05, **P < 0.01, ***P < 0.001)

To evaluate the biocompatibility of Cuf-TMB@PDA, cytotoxicity and hemolysis assays were explored separately. After 24 h of treatment with Cuf-TMB@PDA, a high cell viability rate (89.46%) was observed for mouse L929 cells, even at a concentration as high as 63 µg mL^− 1^, in contrast to cells treated with Cuf-TMB (63.18% viability, Fig. 4a). Furthermore, the proliferation of cells incubated in various leaching solutions with defined concentrations was assessed over a 72-hour period, revealing enhanced cell viability in the presence of Cuf-TMB@PDA leachate. In contrast, cellular activity exhibited a diminished response when exposed to Cuf and Cuf-TMB leachates, indicating the favorable cytocompatibility of the synthesized Cuf-TMB@PDA (Fig. 4b, c). To further assess cytotoxicity, cell viability was evaluated by distinguishing live L929 cells (depicted in green) from deceased cells (represented in red) using calcein-acetoxymethyl (Calcein-AM) and propidium iodide (PI). The majority of L929 cells in the Cuf-TMB@PDA-treated group demonstrated viability, confirming the improved cytocompatibility of Cuf-TMB@PDA (Fig. 4d).

Subsequently, a hemolysis examination was conducted to evaluate the hemocompatibility of the Cuf-TMB@PDA. Upon treatment with Triton, the supernatant of erythrocytes exhibited a distinct red coloration, whereas the supernatants from the Cuf-TMB, Cuf-TMB@PDA, and PBS groups remained transparent and colourless (Fig. S12). These observations indicate the minimal hemolytic effect of the prepared Cuf-TMB@PDA material on erythrocytes. Collectively, these findings underscore the enhanced cytocompatibility and hemocompatibility of Cuf-TMB@PDA, thereby highlighting its potential for in vitro and in vivo wound healing applications when compared to the control groups.

### In vitro antibacterial activity of Cuf-TMB@PDA

**Fig. 5:**
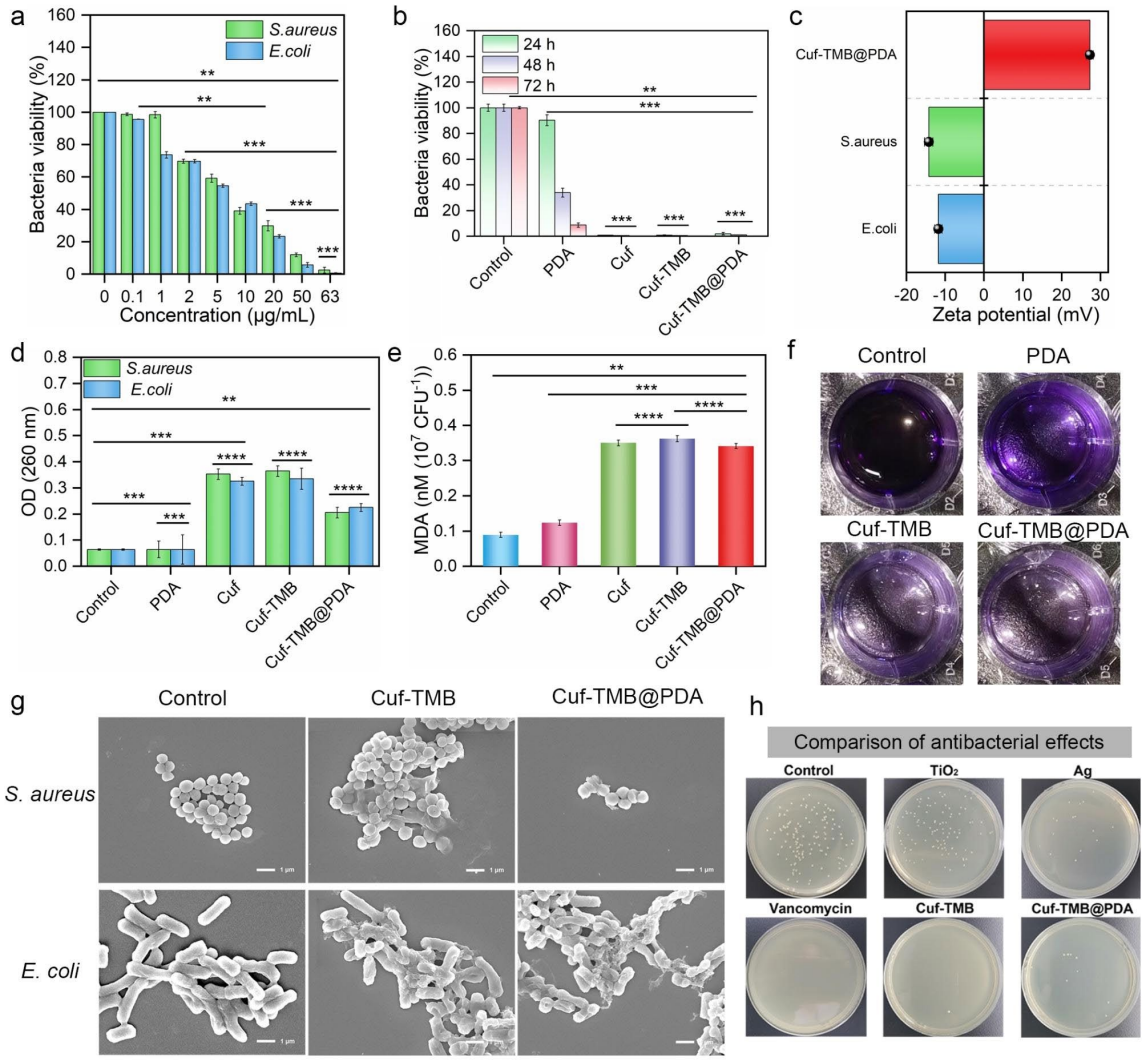
In vitro evaluation of the antimicrobial performance of Cuf-TMB@PDA. (**a**) The inhibitory effect of bacteria after 24-hour incubation in leaching solutions with varying concentrations. (**b**) The inhibition property of *E. coli* after subjecting them to different treatments for 24, 48, and 72 h. (**c**) The zeta potential of Cuf-TMB@PDA in relation to several bacterial zeta potentials. (**d**) OD_260 nm_ of *E. coli* and *S. aureus* treated with PBS, PDA, Cuf, Cuf-TMB, and Cuf-TMB@PDA. (**e**) The MDA level in *E. coli* and *S. aureus* under different treatment conditions. (**f**) Photographs of crystalline violet-stained biofilms after various treatments. (**g**) SEM images of *E. coli* and *S. aureus* incubated with PBS, Cuf-TMB, Cuf-TMB@PDA, respectively. (**h**) The antibacterial ratio of different samples, including TiO_2_, Ag, vancomycin, Cuf-TMB, and Cuf-TMB@PDA, against *E. coli* using the agar plating method (*P < 0.05, **P < 0.01, ***P < 0.001)

The antibacterial efficacy of Cuf-TMB@PDA was firstly evaluated in vitro. At a concentration of 63 µg mL^− 1^, Cuf-TMB@PDA demonstrated significant bactericidal activity against both *E. coli* and *S. aureus*. After 24 h of co-incubation, the survival rate of *E. coli* was reduced to 0.57%, and that of *S. aureus* was reduced to 2.43%. This concentration was determined as the optimal dose of Cuf-TMB@PDA to achieve the desired bactericidal effect (Fig. 5a). Moreover, a bacteriostatic assessment was conducted over a continuous 72-hour period for PBS, PDA, Cuf, Cuf-TMB, and Cuf-TMB@PDA. The PDA treated group showed a gradual emergence of antibacterial activity exhibited by PDA over time. This phenomenon can be attributed to the positive charge on the surface of PDA, which promotes prolonged adherence to bacterial surfaces. Consequently, this disrupts bacterial nutrient absorption, ultimately resulting in bacterial mortality (Fig. 5b, Fig. S13). The concentrations of Cuf and Cuf-TMB were determined based on the copper content in Cuf-TMB@PDA, with a concentration of 6.55 µg mL^− 1^ of Cu(II). Both Cuf and Cuf-TMB demonstrated significant bactericidal efficacy, achieving 99.17% and 99.58% bacterial eradication, respectively, after 24-hour treatment. No bacterial colonies of *E. coli* and *S. aureus* were observed in the Cuf, Cuf-TMB, and Cuf-TMB@PDA groups throughout the 72-hour duration on the agar plate. This observation indicates that both Cuf-TMB and Cuf-TMB@PDA exhibited potent antibacterial activity, which can be attributed to the presence of Cu(II) ions and free radicals.

To further investigate the bactericidal mechanism of Cuf-TMB@PDA, the absorbance values at OD_260 nm_ of various incubation groups were recorded. The OD values of Cuf-TMB@PDA, Cuf, and Cuf-TMB exhibited significantly higher levels compared to those of the control groups. This confirmed the ability of Cuf, Cuf-TMB, and Cuf-TMB@PDA, all containing Cu(II), to induce disruption of the bacterial membrane. Consequently, this led to the release and detectability of its contents (Fig. 5c). In addition, bacterial lipid peroxidation was notably prominent in the Cuf-TMB@PDA, Cuf-TMB, and Cuf-treated groups, which aligns with the previously mentioned antibacterial activities (Fig. 5d).

Bacterial resistance often arises as a result of bacterial biofilm presence, and targeting these biofilms remains an effective strategy for combating bacterial infections. Bacterial biofilms possess the ability to offer protection against external threats, thereby reducing the effectiveness of nanoparticles in antibacterial applications. Therefore, effectively inhibiting biofilm formation has the potential to enhance antibacterial efficacy. To assess the inhibition of biofilm growth, crystal violet staining was employed to quantify the biofilm masses. In all groups containing Cu(II), a noticeable decrease in the rate of biofilm formation was observed, indicating their inhibitory effect on biofilm growth (Fig. 5f, Fig. S14). It is noteworthy to mention that PDA alone exhibited a lesser impact on biofilm formation.

For a deeper understanding of the antimicrobial mechanisms, we conducted SEM measurements to examine the cellular morphology of the bacteria treated with these compounds. In the control-treated groups, the cell membranes of *S. aureus* and *E. coli* remained intact and smooth. However, in the groups treated with Cuf-TMB and Cuf-TMB@PDA, we observed the presence of some noticeable wrinkles on the cell membranes. This observation suggests the antimicrobial effect of Cuf-TMB and Cuf-TMB@PDA in compromising the integrity of bacterial cell membranes (Fig. 5g). Furthermore, certain cell membranes were severely compromised, leading to the release of cytoplasm from within the bacterial cells. Additionally, we performed a comparative analysis to evaluate the antimicrobial properties of traditional antimicrobial materials, including TiO_2_ nanoparticles, Ag nanoparticles, and vancomycin antibiotics. The antimicrobial capabilities demonstrated by both Cuf-TMB@PDA and Cuf-TMB are comparable to antibiotics and significantly surpass the performance of conventional synthetic antimicrobial materials (Fig. 5h, Fig. S15).

In order to investigate the antibacterial mechanism, we utilized ESR to detect free radicals in Cuf-TMB and Cuf-TMB@PDA (Fig. S16). Comparatively, Cuf-TMB@PDA exhibited a diminished intensity of ·OH radicals when compared to Cuf-TMB. This reduction in intensity can be attributed to the depletion of radicals during the polymerization process of dopamine into polydopamine. The presence of residual free radicals in the Cuf-TMB@PDA system was confirmed through ESR analysis. These free radicals have the potential to induce peroxidation within the bacterial cells, augmenting the antimicrobial effect by synergizing with Cu(II) and exerting a bactericidal effect. These results validate the remarkable bactericidal properties and superior biocompatibility of the antibacterial platform, Cuf-TMB@PDA, making it a promising candidate for in vivo applications.

### In vivo wound healing of Cuf-TMB@PDA

**Fig. 6:**
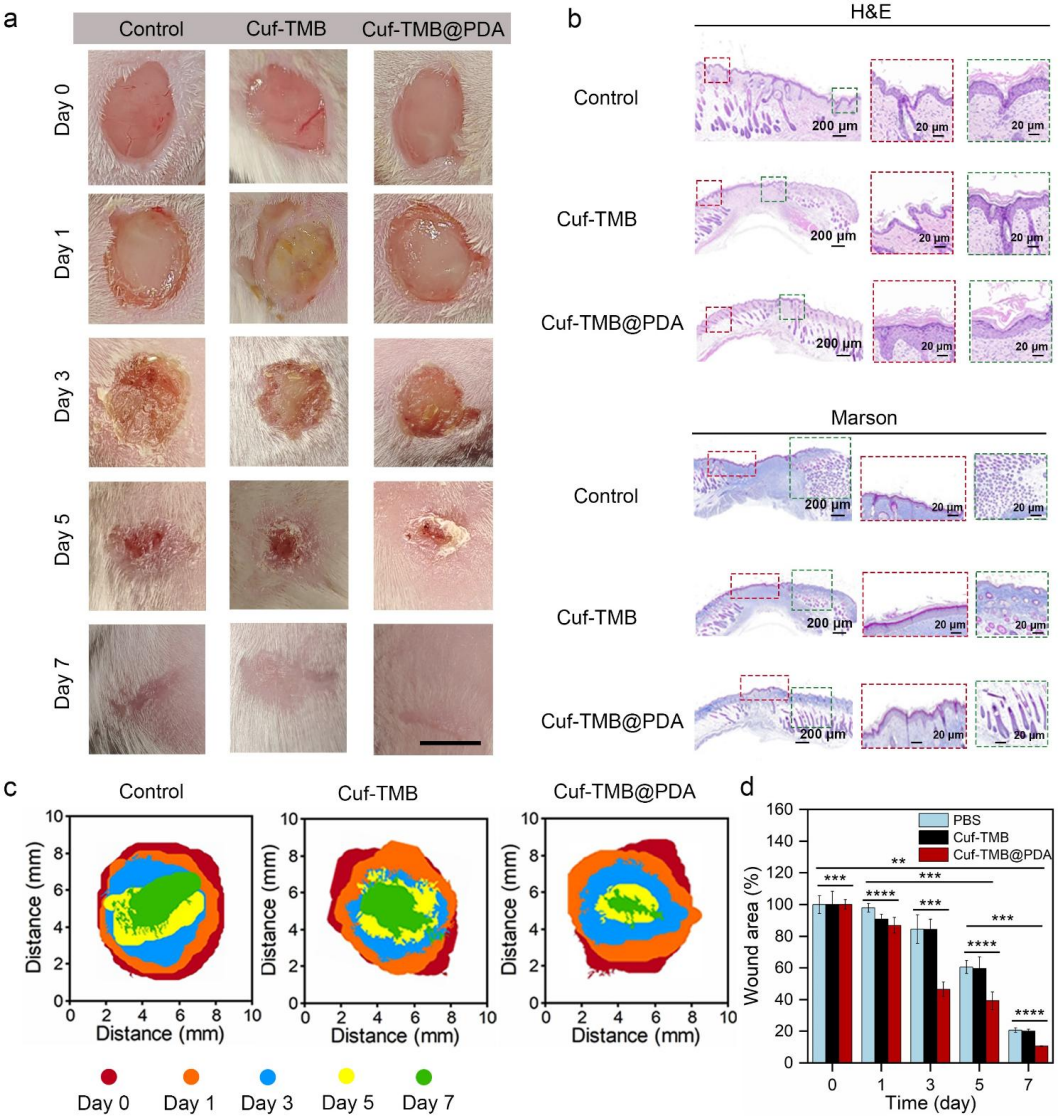
In vivo wound closure assessment of Cuf-TMB@PDA. (**a**) Photographs captured at various time points (0, 1, 3, 5, and 7 days) to visually document the progress of wound healing with different dressings. Each photograph was accompanied by a 10 mm scale bar. (**b**) H&E stained sections analyzed on the 7th day of the wound healing models. The red box represents the newly formed granulation tissue, while the green box indicates the presence of hair follicles. (**c**) Masson’s trichrome stained sections were utilized to detect collagen (shown as blue staining). (**d**) Schematic images of wound contraction during 7 days for the treatments by PBS, Cuf-TMB, and Cuf-TMB@PDA. Wound area for each group (n = 3). (**e**) Corresponding wound area recorded at various time points to assess the progression of healing (*P < 0.05, **P < 0.01, ***P < 0.001)

To further validate the in vivo antibacterial efficacy, a mouse wound infection model was employed as a proof of concept. The mice were divided into three groups, each receiving different treatments: (1) PBS; (2) Cuf-TMB; and (3) Cuf-TMB@PDA. The antimicrobial effect was evaluated by monitoring wound size at 1, 3, 5, and 7 days, along with the quantification of bacterial counts within the wounds (Fig. 6a). Additionally, the presence of bacteria at the wound sites was assessed using the agar plate method to determine the bactericidal effect Fig. S17). As anticipated, both the Cuf-TMB and Cuf-TMB@PDA groups exhibited minimal bacterial growth on the agar plates, which aligned with the results obtained from the in vitro experiments. Schematic illustrations depicting wound contraction and the calculation of wound contraction were also provided in Fig. 6b. Notably, after 7 days of treatment, the wound treated with Cuf-TMB@PDA presented the smallest proportion of open wound area (10.63%) and displayed a smoother appearance with the presence of newly formed epidermal and dermal tissues. In contrast, the Cuf-TMB and PBS groups exhibited open wound areas of 19.99% and 20.79%, respectively, accompanied by visibly uneven scar tissue. These findings strongly highlight the superior wound healing efficacy of the Cuf-TMB@PDA group compared to the other treatment groups.

**Fig. 7:**
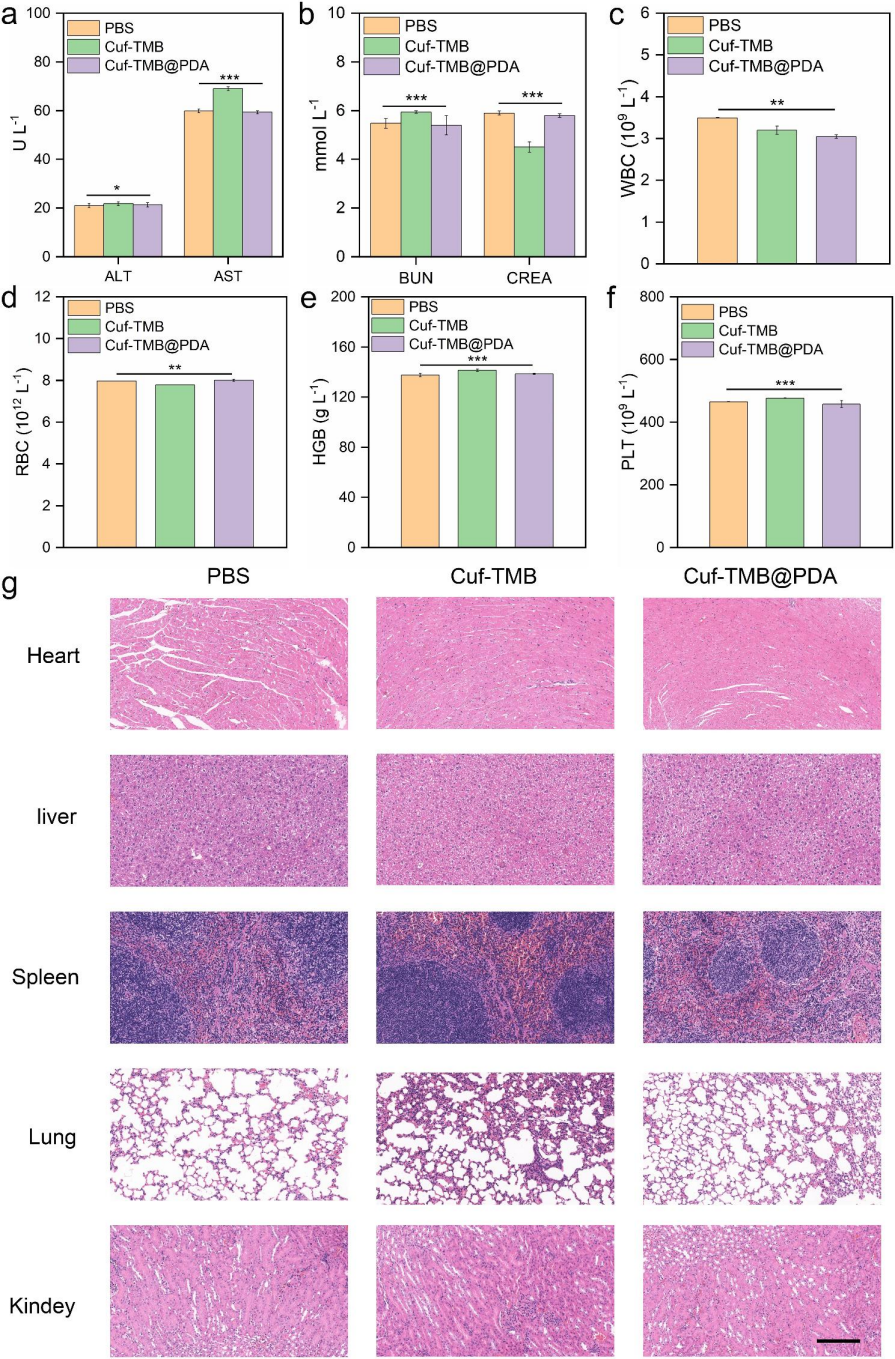
Biosafety evaluation of Cuf-TMB@PDA. (**a**) Liver function test of alanine aminotransferase (ALT) and aspartate aminotransferase (AST). (**b**) Kidney function test of blood urea nitrogen (BUN) and creatinine (CREA). (**c**)-(**f**) Blood analysis of mice after exposing to different materials after 7 days, respectively. (**c**) Total white blood cell (WBC), (**d**) total red blood cells (RBC), (**e**) hemoglobin (HGB) and (**f**) blood platelet (PLT) counts (*P < 0.05, **P < 0.01, ***P < 0.001). (**g**) H&E staining of the heart, liver, spleen, lung, and kidney with different treatments after 7 days (scale bar: 200 μm)

To affirm the observed healing outcomes, a comprehensive evaluation of wound healing was conducted by examining histological changes in the skin tissue. Upon completion of the treatment period, the mice were sacrificed at specific time intervals, and tissue samples were collected for analysis. H&E staining, as well as Masson staining, were utilized to evaluate the in vivo wound healing efficacy of Cuf-TMB and Cuf-TMB@PDA. At the 7th day, a notable abundance of granulation tissue was observed in the Cuf-TMB@PDA group, surpassing the other groups (Fig. 6c). The H&E staining results revealed that the scar width in the Cuf-TMB@PDA-treated group (1.15 mm) was significantly smaller compared to the PBS (5.59 mm) and Cuf-TMB (1.52 mm) groups. Additionally, upon closer examination of the H&E images, the Cuf-TMB@PDA-treated skin tissue exhibited reduced inflammatory signals (blood cells and neutrophils), increased collagen fibers, and visible dermal tissue, including hair follicle appendages, among other characteristics. During the maturation phase of wound repair, collagen deposition plays a critical role in scar formation, while wound contraction is facilitated by the interconnection of fibroblasts and collagen. The accumulation of newly formed collagen in the regenerated skin tissue was evident through Masson staining. Notably, the Cuf-TMB@PDA group demonstrated elevated levels of collagen deposition at the wound edges compared to the control group. Similarly, the analysis of kidney and liver function did not demonstrate any toxicity within the organism (Fig. 7a and b). Other blood parameters, such as white blood cell (WBC), red blood cells (RBC), hemoglobin (HGB) and blood platelet (PLT), fell within the normal range (Fig. 7c and f). Moreover, H&E staining revealed that Cuf-TMB@PDA did not induce any damage or toxicity to the normal anatomical structures of diverse organs throughout the treatment duration (Fig. 7a g). Hence, Cuf-TMB@PDA treatment has no toxic effect on the circulatory system of organisms. All these results strongly supported that the potential of Cuf-TMB@PDA as a promising candidate for wound healing purposes.

## Conclusions

To summarize, our study introduced an antimicrobial agent called Cuf-TMB@PDA, which was applied to investigate the healing of skin wounds in mice. Unlike previously reported copper-based antimicrobial materials that exhibited significant cytotoxicity, Cuf-TMB@PDA demonstrated enhanced antibacterial efficacy without compromising cytocompatibility. The positive charge of Cuf-TMB@PDA enables it to effectively trap and interact with negatively charged bacteria through electrostatic adsorption. This close interaction exposes the bacteria to the encapsulated free radicals within Cuf-TMB@PDA, leading to oxidative damage and the concurrent release of Cu(II) within an optimal range. These combined mechanisms greatly enhance the antibacterial effectiveness of Cuf-TMB@PDA. Importantly, our findings indicate that Cuf-TMB@PDA exhibits low toxicity in complex settings. With its dual attributes of antimicrobial effectiveness and cytocompatibility, Cuf-TMB@PDA holds significant potential for applications in skin tissue repair, providing inherent anti-infective protection.

### Electronic supplementary material

Below is the link to the electronic supplementary material.

**Supplementary Material 1**: **Experimental Section; Scheme S1**. Dopamine undergoes polymerization in the presence of free radicals, resulting in the formation of polydopamine. **Fig. S1**. The FT-IR spectra of Cuf-TMB@PDA and Cuf-TMB. **Fig. S2**. Zeta potentials of Cuf-TMB@PDA NPs using different concentrations of dopamine. **Fig. S3**. XRD pattern of Cuf-TMB@PDA and Cuf-TMB. **Fig. S4**. (a) The SAED pattern of Cuf-TMB NPs. (b) HRTEM image of Cuf-TMB NPs. (c) The SAED pattern of Cuf-TMB@PDA. (d) HRTEM image of Cuf-TMB@PDA. **Fig. S5**. SEM images of (a) Cuf-TMB and (b) Cuf-TMB@PDA. **Fig. S6**. The Energy Dispersive X-Ray Spectroscopy (EDX) mapping of Cuf-TMB. **Fig. S7**. Fine XPS spectra of Cuf-TMB NPs: (a) Cu 2p; (b) C 1s; (c) N 1s; (d) O 1s. **Fig. S8**. The Brunauer-Emmett-Teller (BET) characterization of Cuf-TMB@PDA and Cuf-TMB. **Fig. S9**. (a) Reaction-time curves of TMB colorimetric reactions catalyzed by Cuf-TMB@PDA. (b) Comparison of the specific activities of Cuf-TMB@PDA using different concentrations of dopamine. **Fig. S10**. (a)-(g) Comparison of particle size for Cuf-TMB coated with varying concentrations of dopamine. **Fig. S11**. (a) Effect of pH value on the POD-like activity of Cuf-TMB@PDA. (b) Effect of temperature on the POD-like activity of Cuf-TMB@PDA. **Fig. S12**. (a) Evaluation of hemocompatibility of different concentrations of Cuf-TMB@PDA. (b) Hemocompatibility Evaluation, Triton X-100, PBS, Cuf-TMB, Cuf-TMB@PDA. **Fig. S13**. Comparison of the inhibition effect of Cuf-TMB@PDA acting on bacteria (*E. coli* and *S. aureus*). **Fig. S14**. The growth curves of (a) *E. coli* and (b) *S. aureus* after incubation with different concentrations (from 0 to 63 μg mL^-1^) of Cuf-TMB@PDA. **Fig. S15**. Evaluation of the antimicrobial activities of TiO2, Ag, vancomycin antibiotic, Cuf-TMB, and Cuf-TMB@PDA. **Fig. S16**. The ESR spectra of Cuf-TMB@PDA and Cuf-TMB. **Fig. S17**. Representative photographs of bacterial cultures taken from *S. aureus* infected wound areas at different times during the treatment phase. **Fig. S18**. Dynamic body weight changes of *S. aureus* infected rats in various groups over 7 days. **Tab. S1**. Analysis of FTIR spectra of Cuf-TMB@PDA NPs. **Tab. S2**. Elemental analysis of Cuf-TMB and Cuf-TMB@PDA.

## Data Availability

All data generated or analyzed during this are included in this paper.
