# Supplementary material for: Biocompatible copper formate-based nanoparticles with strong antibacterial properties for wound healing
Source: J Nanobiotechnology. 2023 Dec 10;21:474. doi: 10.1186/s12951-023-02247-w (PMC10710715; doi:10.1186/s12951-023-02247-w)
Supplement: Supplementary file 1 — Supplementary Material 1: Experimental Section; Scheme S1. Dopamine undergoes polymerization in the presence of free radicals, resulting in the formation of polydopamine. Fig. S1. The FT-IR spectra of Cuf-TMB@PDA and Cuf-TMB. Fig. S2. Zeta potentials of Cuf-TMB@PDA NPs using different concentrations of dopamine. Fig. S3. XRD pattern of Cuf-TMB@PDA and Cuf-TMB. Fig. S4. (a) The SAED pattern of Cuf-TMB NPs. (b) HRTEM image of Cuf-TMB NPs. (c) The SAED pattern of Cuf-TMB@PDA. (d) HRTEM image of Cuf-TMB@PDA. Fig. S5. SEM images of (a) Cuf-TMB and (b) Cuf-TMB@PDA. Fig. S6. The Energy Dispersive X-Ray Spectroscopy (EDX) mapping of Cuf-TMB. Fig. S7. Fine XPS spectra of Cuf-TMB NPs: (a) Cu 2p; (b) C 1s; (c) N 1s; (d) O 1s. Fig. S8. The Brunauer-Emmett-Teller (BET) characterization of Cuf-TMB@PDA and Cuf-TMB. Fig. S9. (a) Reaction-time curves of TMB colorimetric reactions catalyzed by Cuf-TMB@PDA. (b) Comparison of the specific activities of Cuf-TMB@PDA using different concentrations of dopamine. Fig. S10. (a)-(g) Comparison of particle size for Cuf-TMB coated with varying concentrations of dopamine. Fig. S11. (a) Effect of pH value on the POD-like activity of Cuf-TMB@PDA. (b) Effect of temperature on the POD-like activity of Cuf-TMB@PDA. Fig. S12. (a) Evaluation of hemocompatibility of different concentrations of Cuf-TMB@PDA. (b) Hemocompatibility Evaluation, Triton X-100, PBS, Cuf-TMB, Cuf-TMB@PDA. Fig. S13. Comparison of the inhibition effect of Cuf-TMB@PDA acting on bacteria (E. coli and S. aureus). Fig. S14. The growth curves of (a) E. coli and (b) S. aureus after incubation with different concentrations (from 0 to 63 μg mL-1) of Cuf-TMB@PDA. Fig. S15. Evaluation of the antimicrobial activities of TiO2, Ag, vancomycin antibiotic, Cuf-TMB, and Cuf-TMB@PDA. Fig. S16. The ESR spectra of Cuf-TMB@PDA and Cuf-TMB. Fig. S17. Representative photographs of bacterial cultures taken from S. aureus infected wound areas at different times during the treatment phase. Fig. S18. Dynami [file 12951_2023_2247_MOESM1_ESM.docx]

**Supporting Information**

Biocompatible copper formate-based nanoparticles with strong antibacterial properties for wound healing

Yue Zhou^a, b^, Ping Sun^a, b^, Yongbin Cao^a, b^, Jiahao Yang^b, c^, Qingzhi Wu^a, b, c^ *, Jian Peng^a, b, c^ *

^a^ State Key Laboratory of Advanced Technology for Materials Synthesis and Processing, Wuhan 430070, China

^b^ School of Chemistry, Chemical Engineering and Life Science, Wuhan University of Technology, Wuhan 430070, China

^c^ School of Material Science and Engineering, Wuhan University of Technology, Wuhan 430070, China

Corresponding authors: [wuqzh@whut.edu.cn](mailto:wuqzh@whut.edu.cn); jianpeng@whut.edu.cn

*Experimental Section*

*1. Optimize the concentration of dopamine*

The concentration of dopamine was varied from 10^-5^ to 10^3^ μg mL^-1^ to investigate the effect of concentration on the production of the Cuf-TMB@PDA. The Zeta potentials were measured to compare the different concentrations of dopamine-coated Cuf-TMB. Simultaneously, UV-vis spectroscopy was employed to authenticate the peroxidase-like (POD-like) activity of the diverse concentrations coated onto Cuf-TMB.

*2. Hematocompatibility Evaluation*

The hemolysis test was performed according to previously described guidelines [1]. The cardiac aorta of healthy BALB/c female mice was used to get fresh whole blood samples. The collected blood samples were centrifuged at 3500 rpm for 15 minutes to collect red blood cells and then gently washed three times with saline. Then, 50 µL of the erythrocyte suspension was mixed with 950 µL of different concentrations of Cuf-TMB@PDA dispersion (0.05-5 µg mL^-1^). The mixture was incubated at 37°C for 3 hours. Hemolysis was measured using a microplate reader to collect the supernatant's absorbance at 540 nm. Triton X-100 and phosphate buffered saline (PBS) were used as the positive and negative controls, respectively.

*3. Histologic analysis*

To evaluate epidermal regeneration and wound healing, the harvested skin samples were first fixed in 4% paraformaldehyde for a duration of 1 hour. Subsequently, the samples were embedded in paraffin and cross-sectioned into 4-μm-thick slices. These sections were further subjected to staining with hematoxylin-eosin and Masson's trichrome stain. The analysis and documentation of all sections were carried out using microscopy.

*4. In vivo antibacterial assay*

In brief, female Babl/c mice weighing 18-22 g were anesthetized with 5 wt% chloral hydrate and securely positioned on a surgical corkboard. Following skin disinfection through iodine shaving, two incisions measuring 7 mm-diameter each were made on the dorsal surface of the mice. Subsequently, 10 μL of Staphylococcus aureus (10^8^ CFU mL^-1^) was injected into the wounds to induce infection. The well-being of the animals and the progression of wound infection were continuously observed, documented, and photographed throughout the duration of the study, up to the point where noticeable infection lesions formed at the wound site.

Periodically, tissue fluid samples were collected from the wounds using sterile cotton swabs, followed by dilution and application onto LB agar plates. The colonies were enumerated post-incubation in a constant-temperature incubator. This experiment was repeated three times for each group.


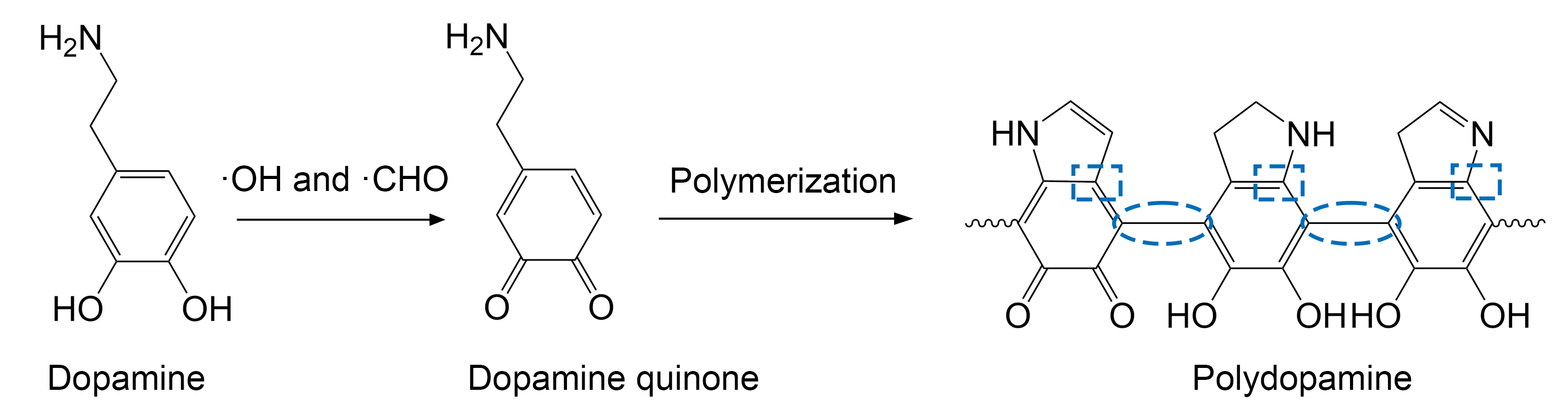


**Scheme S1.** Dopamine undergoes polymerization in the presence of free radicals, resulting in the formation of polydopamine.





**Fig. S1. The FT-IR spectra of Cuf-TMB@PDA and Cuf-TMB.** There was also no significant change observed in the IR peaks of Cuf-TMB@PDA, presumably attributed to the fact that the TMB in Cuf-TMB also contains functional groups such as amine. Similar to the amino functional groups in PDA in Cuf-TMB@PDA.

**Tab. S1.** Analysis of FTIR spectra of Cuf-TMB@PDA NPs.

| Wavenumbers (cm^-1^) | Functional groups | Ref. |
| --- | --- | --- |
| 3388, 2922 | O-H stretching | [2] |
| 1633 | -C(=O)H | [3] |
| 1472 | *δ*_a_(CH_3_) | [4] |
| 1380 | *ν*_s_(C-N) | [5] |
| 1277 | *δ*(C-H) | [6] |
| 862 | C–H deformation of phenyl ring | [7] |





**Fig. S2**. Zeta potentials of Cuf-TMB NPs with different concentrations of dopamine. The concentration of dopamine was optimized to be 0.1 μg mL^-1^.





**Fig. S3.** XRD pattern of Cuf-TMB@PDA and Cuf-TMB.


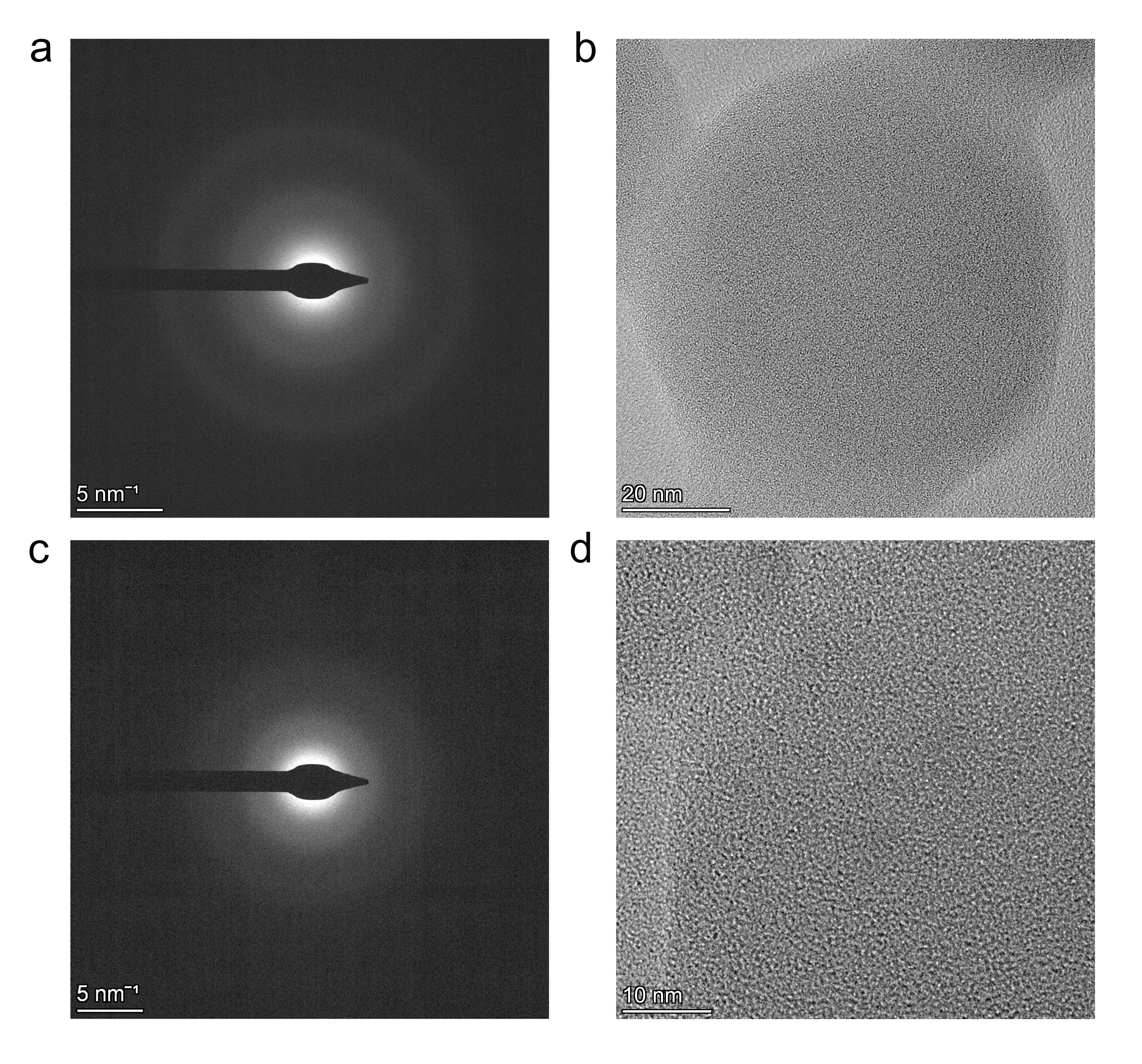


**Fig. S4. (a) The SAED pattern of Cuf-TMB NPs. (b) HRTEM image of Cuf-TMB NPs. (c) The SAED pattern of Cuf-TMB@PDA. (d) HRTEM image of Cuf-TMB@PDA.** No significant alterations were detected in the diffraction rings and lattice stripes, indicating that both Cuf-TMB and Cuf-TMB@PDA maintained their amorphous nature.


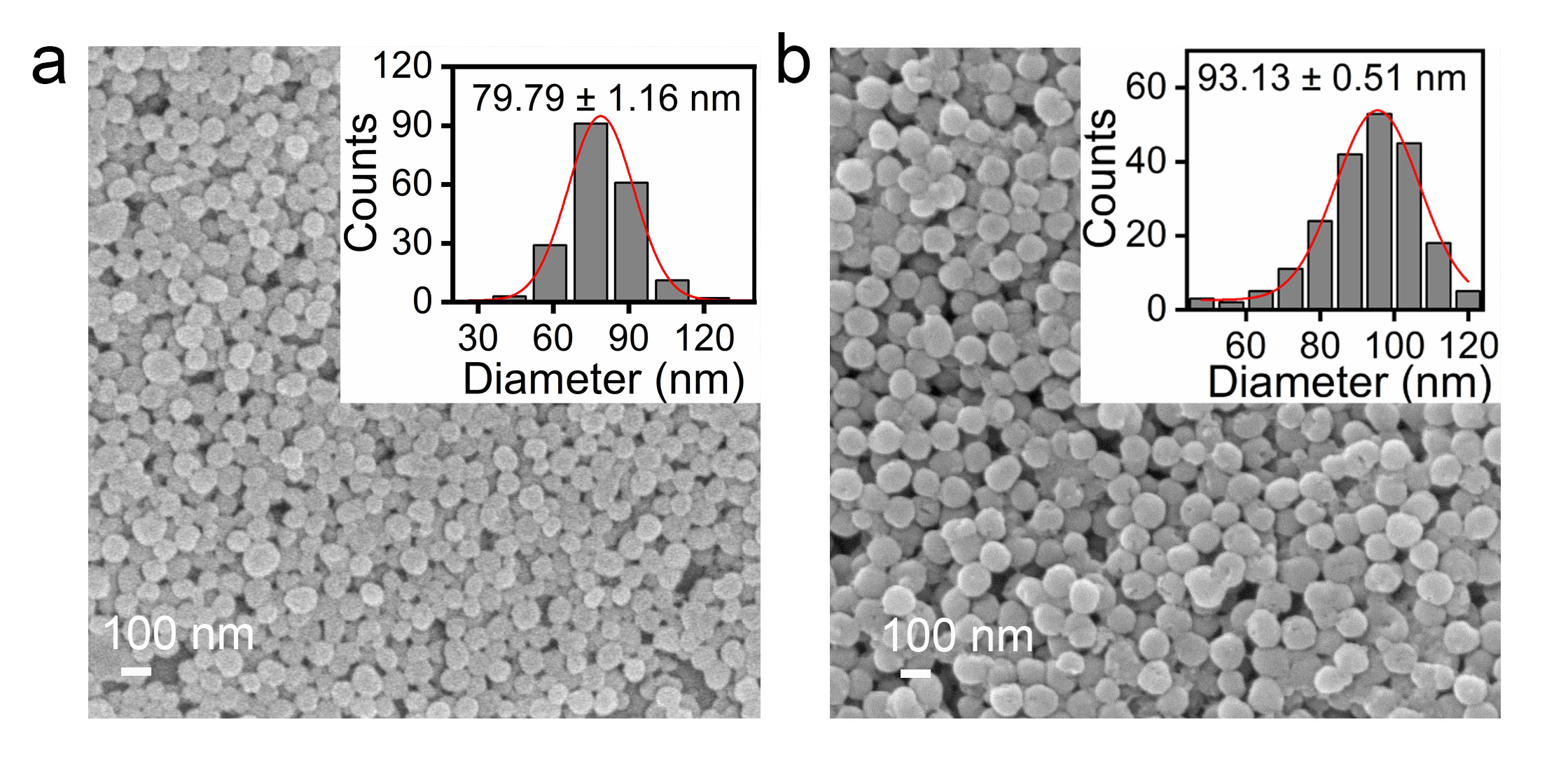


**Fig. S5. SEM images of (a) Cuf-TMB and (b) Cuf-TMB@PDA.** Scale bar: 100 nm. SEM images indicate that the morphology and size of Cuf-TMB@PDA appear as spherical with a diameter of 93.13 nm. The particle size of Cuf-TMB was approximately 79.19 nm, and this size difference provides evidence of the presence of the PDA layer coating.


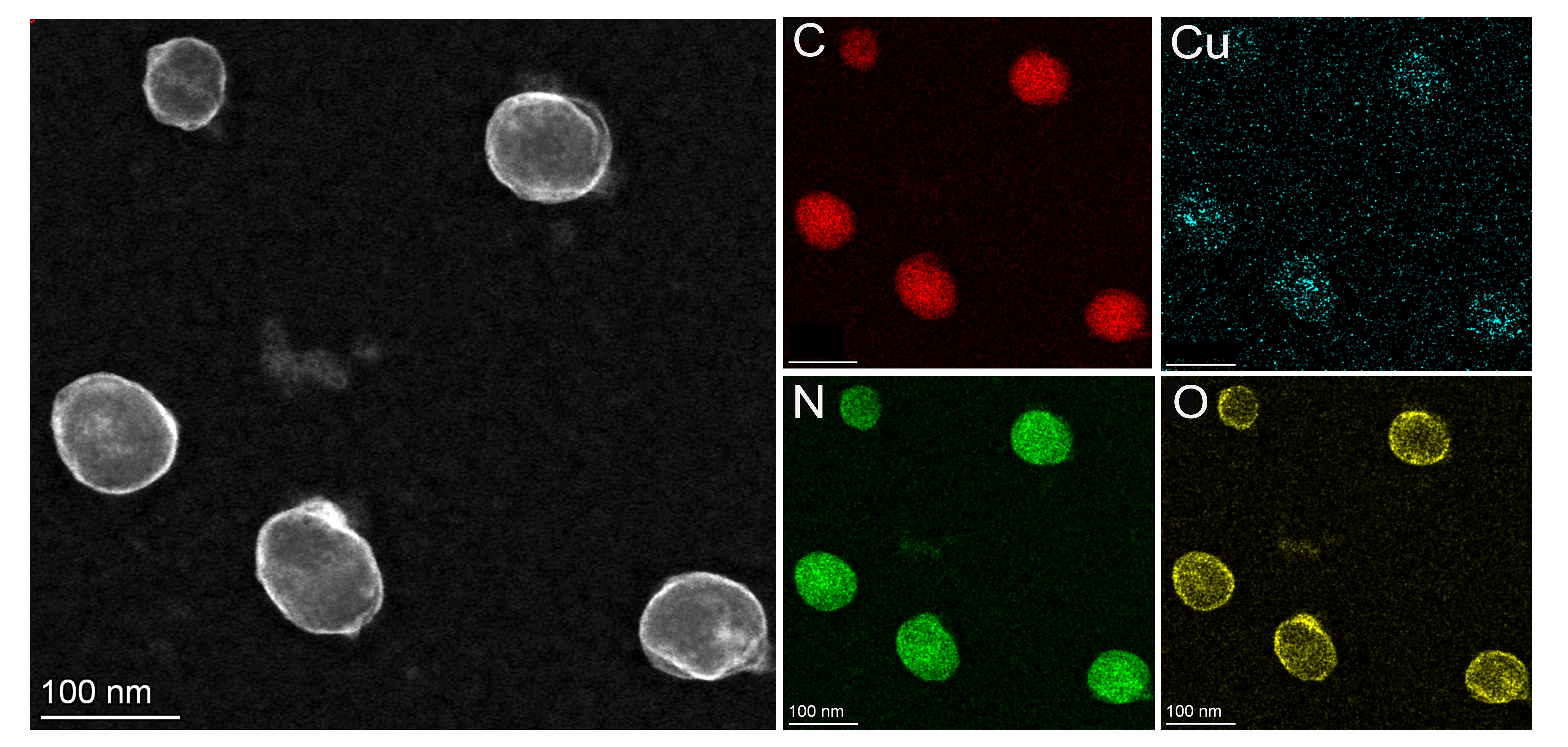


**Fig. S6.** The Energy Dispersive X-Ray Spectroscopy (EDX) mapping of Cuf-TMB. Scale bar: 100 nm.

**Table S2.** **Elemental analysis of Cuf-TMB and Cuf-TMB@PDA.**

| Elemental composition | Cuf-TMB | Cuf-TMB@PDA |
| --- | --- | --- |
| Cu | 8.59% | 1.04% |
| N | 6.41% | 23.89% |
| O | 21.82% | 24.63% |
| C | 57.16% | 46.56% |
| H | 6.03% | 3.88% |


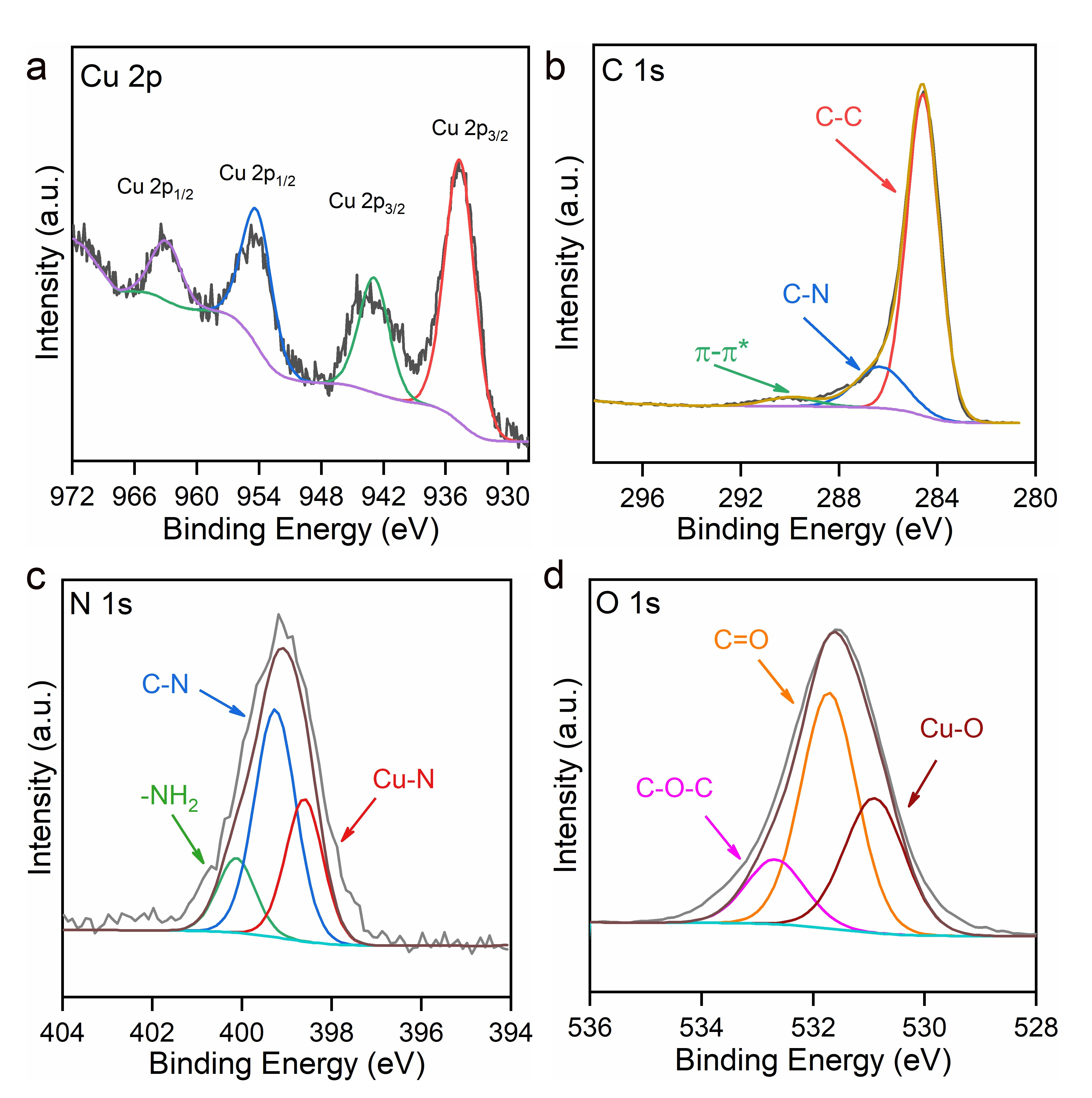


**Fig. S7.** **Fine XPS spectra of Cuf-TMB NPs: (a) Cu 2p; (b) C 1s; (c) N 1s; (d) O 1s.**





**Fig. S8. The Brunauer-Emmett-Teller (BET) characterization of Cuf-TMB@PDA and Cuf-TMB.** The specific surface area of Cuf-TMB@PDA is 101.01 cm² g^-1^, in contrast to the 44.13 cm² g^-1^ observed for Cuf-TMB alone.


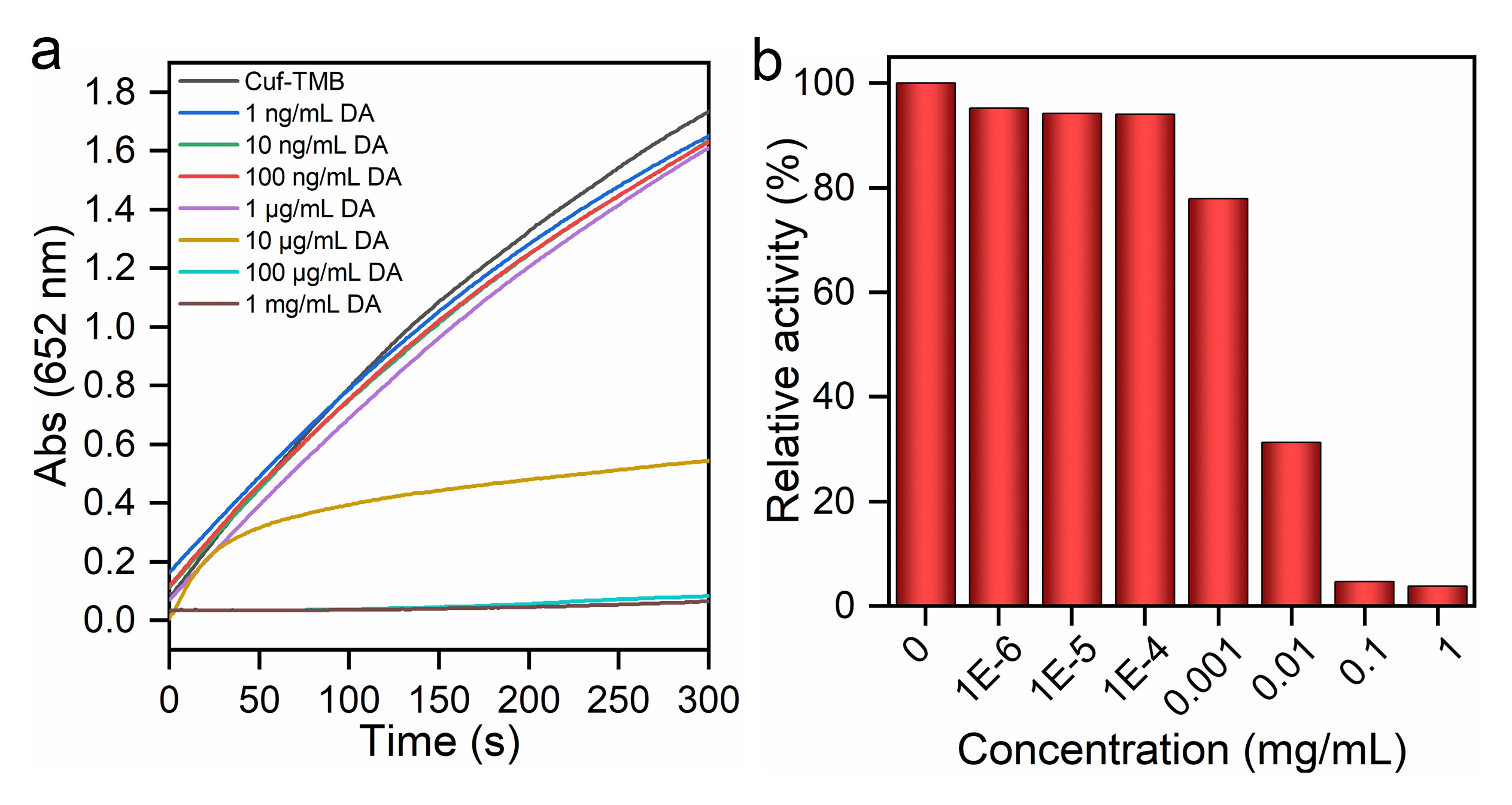


**Fig. S9.** **(a) Reaction-time curves of TMB colorimetric reactions catalyzed by Cuf-TMB@PDA. (b) Comparison of the specific activities of Cuf-TMB@PDA with different concentrations of dopamine.** To verify the appropriate concentrations of dopamine, the POD-like activity of Cuf-TMB@PDA using different dopamine concentrations were compared. It was demonstrated that the optimal dopamine concentration is 0.1 μg mL^-1^.


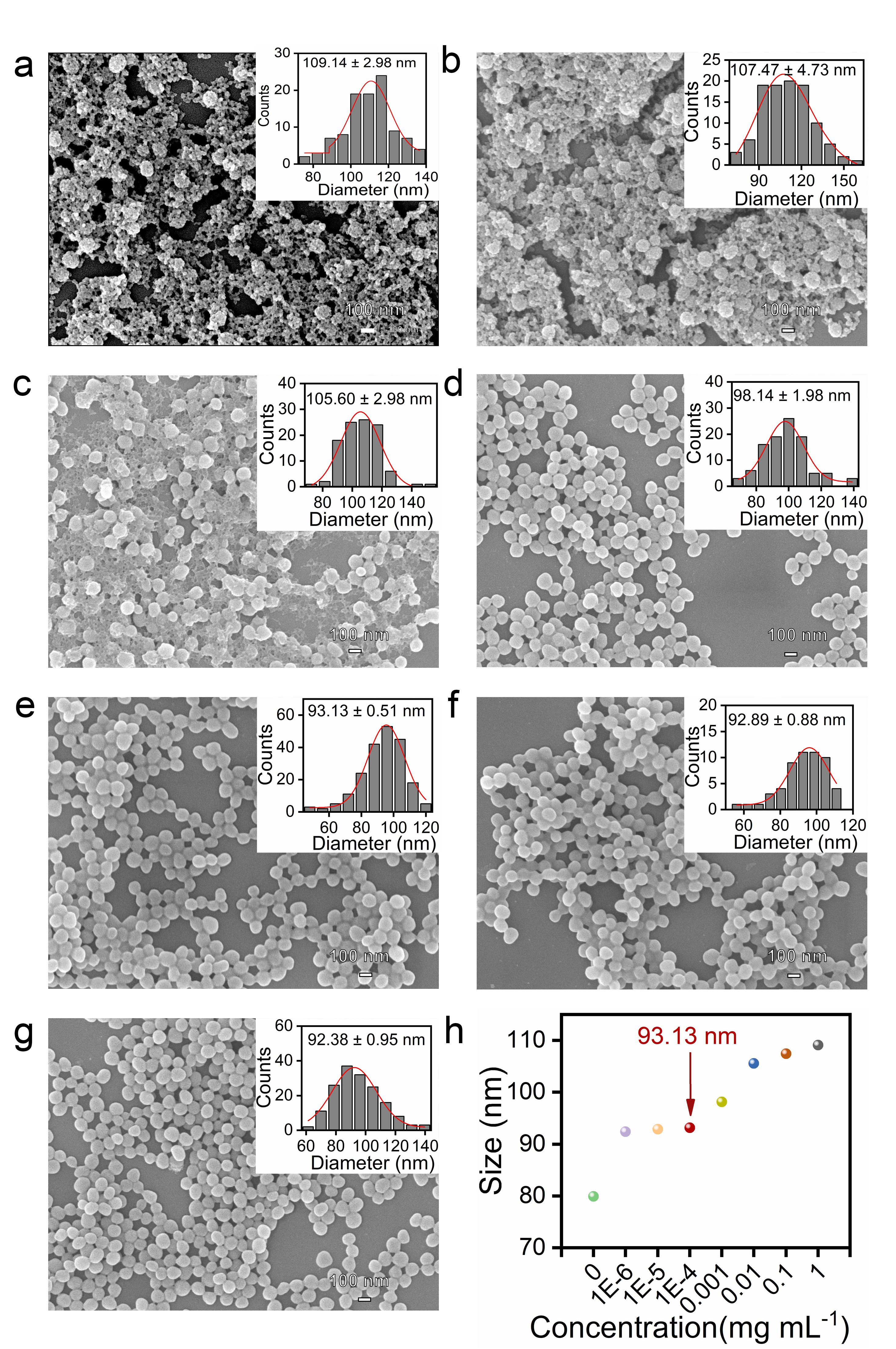


**Fig. S10.** **(a)-(g) Comparison of particle size for Cuf-TMB coated with varying concentrations of dopamine.** The dopamine concentrations from (a)-(g) are 1000 μg mL^-1^, 100 μg mL^-1^, 10 μg mL^-1^, 1 μg mL^-1^, 0.1 μg mL^-1^, 0.01 μg mL^-1^, and 0.001 μg mL^-1^, respectively. The particle size remains at ~93.13 nm for dopamine concentrations less than or equal to 0.1 μg mL^-1^.


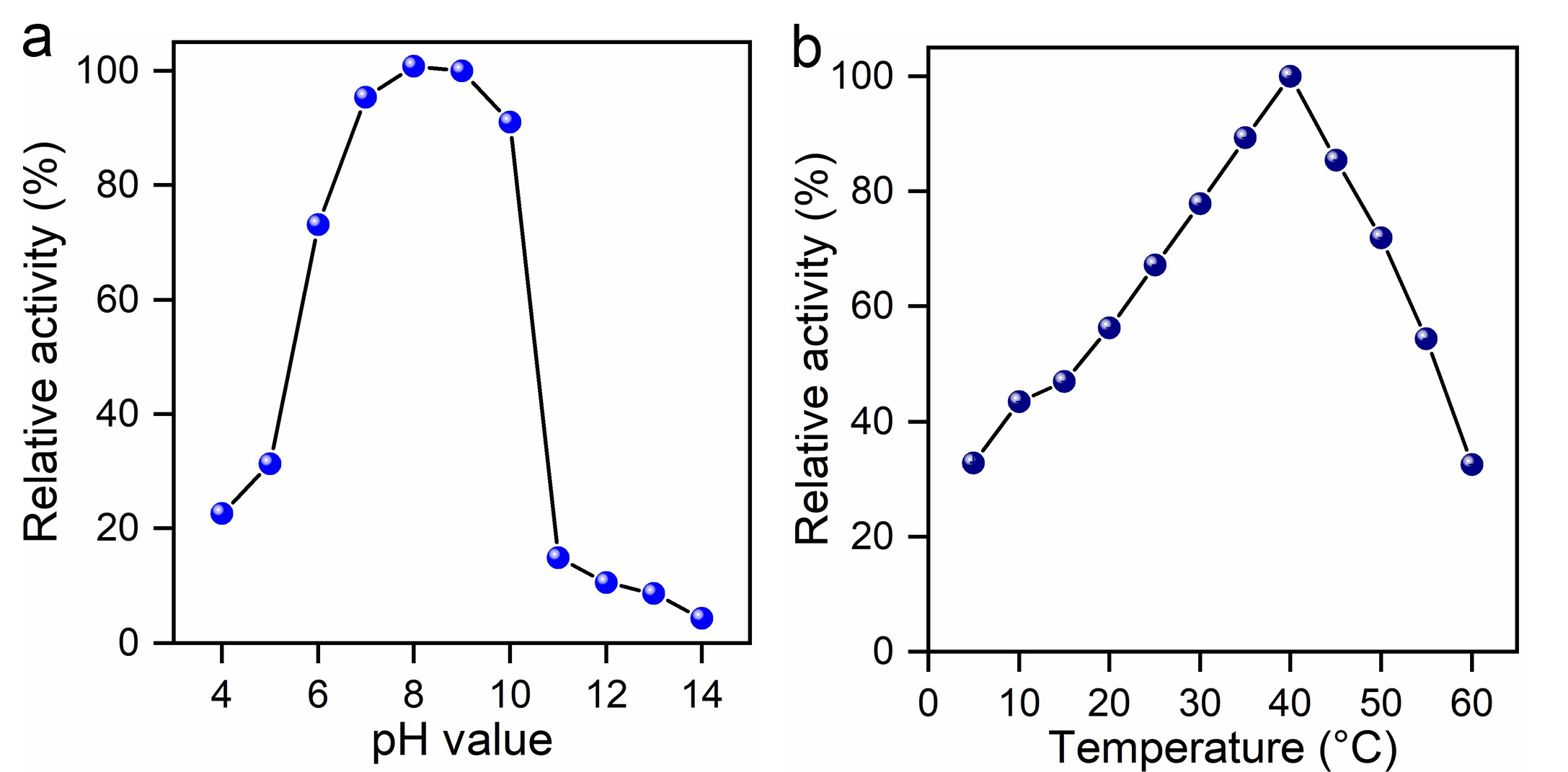


**Fig. S11. (a) Effect of pH value on the POD-like activity of Cuf-TMB@PDA. The POD-like activity of Cuf-TMB@PDA was evaluated at different pHs, which shows good performance at pH 7. (b) Effect of temperature on the POD-like activity of Cuf-TMB@PDA.** The absorbance at 652 nm shows a peak at 40 ℃ within a temperature range of 5 ℃ to 60 ℃. Thus, 40 ℃ is selected as the optimal temperature during the experiment.


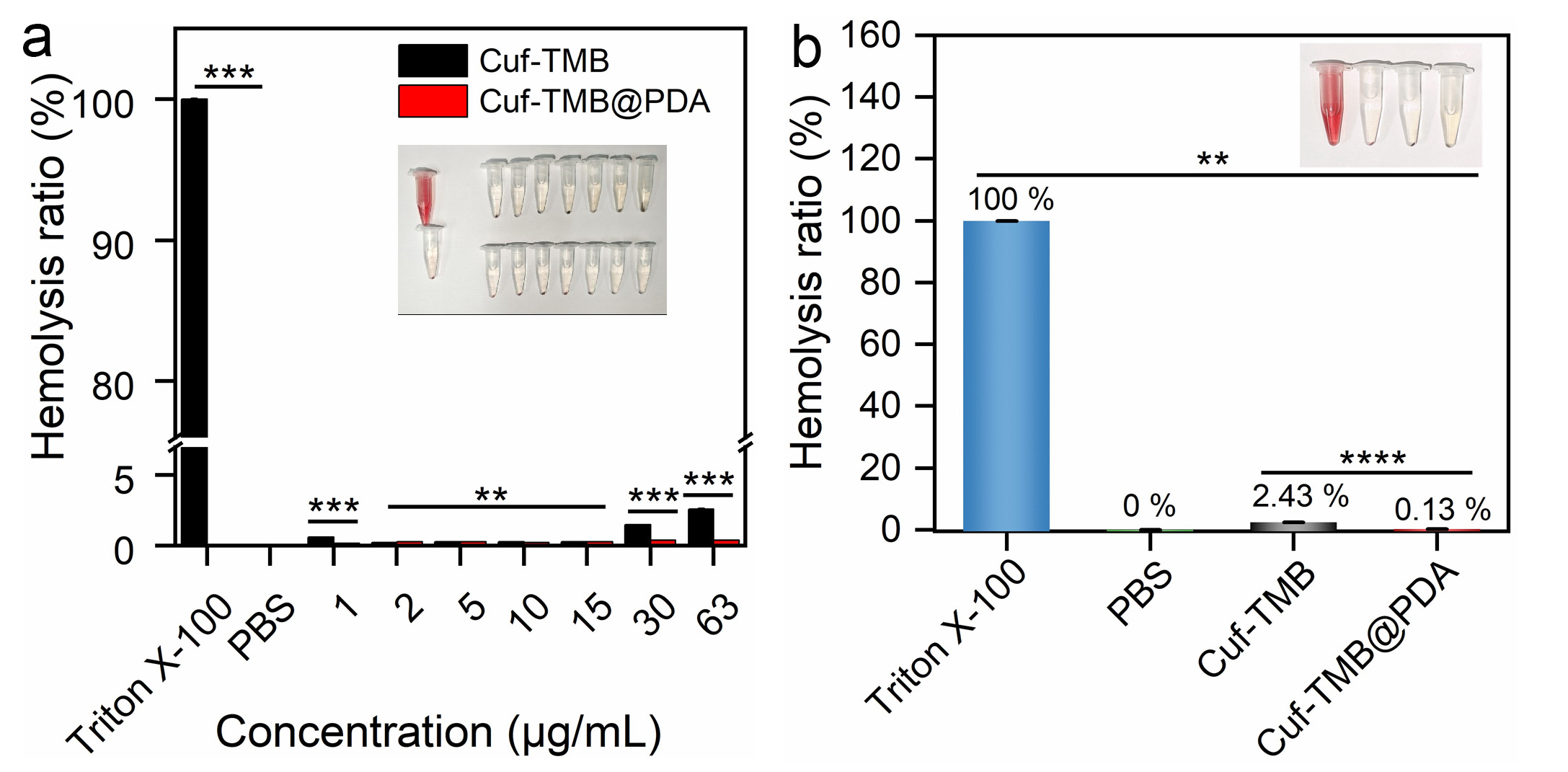


**Fig. S12.** **(a) Evaluation of the hemocompatibility for different concentrations of Cuf-TMB@PDA. (b) Hemocompatibility comparison of Triton X-100, PBS, Cuf-TMB and Cuf-TMB@PDA.** Hemolysis control experiments were conducted for various material treatments, revealing that Cuf-TMB@PDA demonstrated hemocompatibility with erythrocytes comparable to that of the PBS treatment (**P* < 0.05, ***P* < 0.01, ****P* < 0.001).


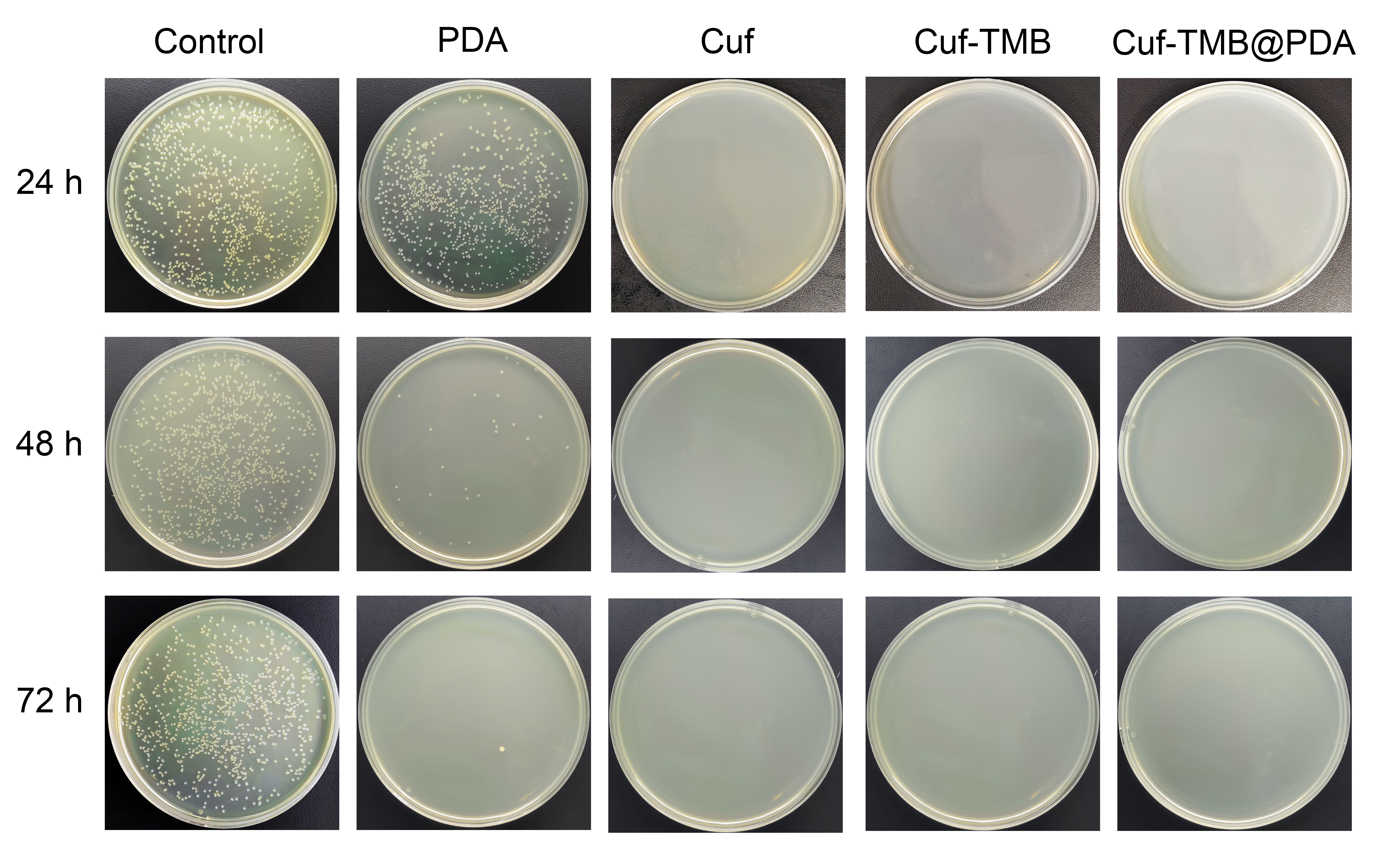


**Fig. S13.** **Comparison of the inhibition effect of Cuf-TMB@PDA acting on bacteria (*E. coli* and *S. aureus*)*.*** The number of bacterial colony-forming units (CFUs) in the blank group showed no obvious difference, implying that the PBS cannot impede the normal growth of *E. coli* and *S. aureus*. Regarding the group treated with PDA, it was observed that PDA exhibited a gradual antimicrobial effect over time. This phenomenon could be attributed to the positively charged surface of PDA, which has the capability to adhere to bacterial surfaces for extended periods.


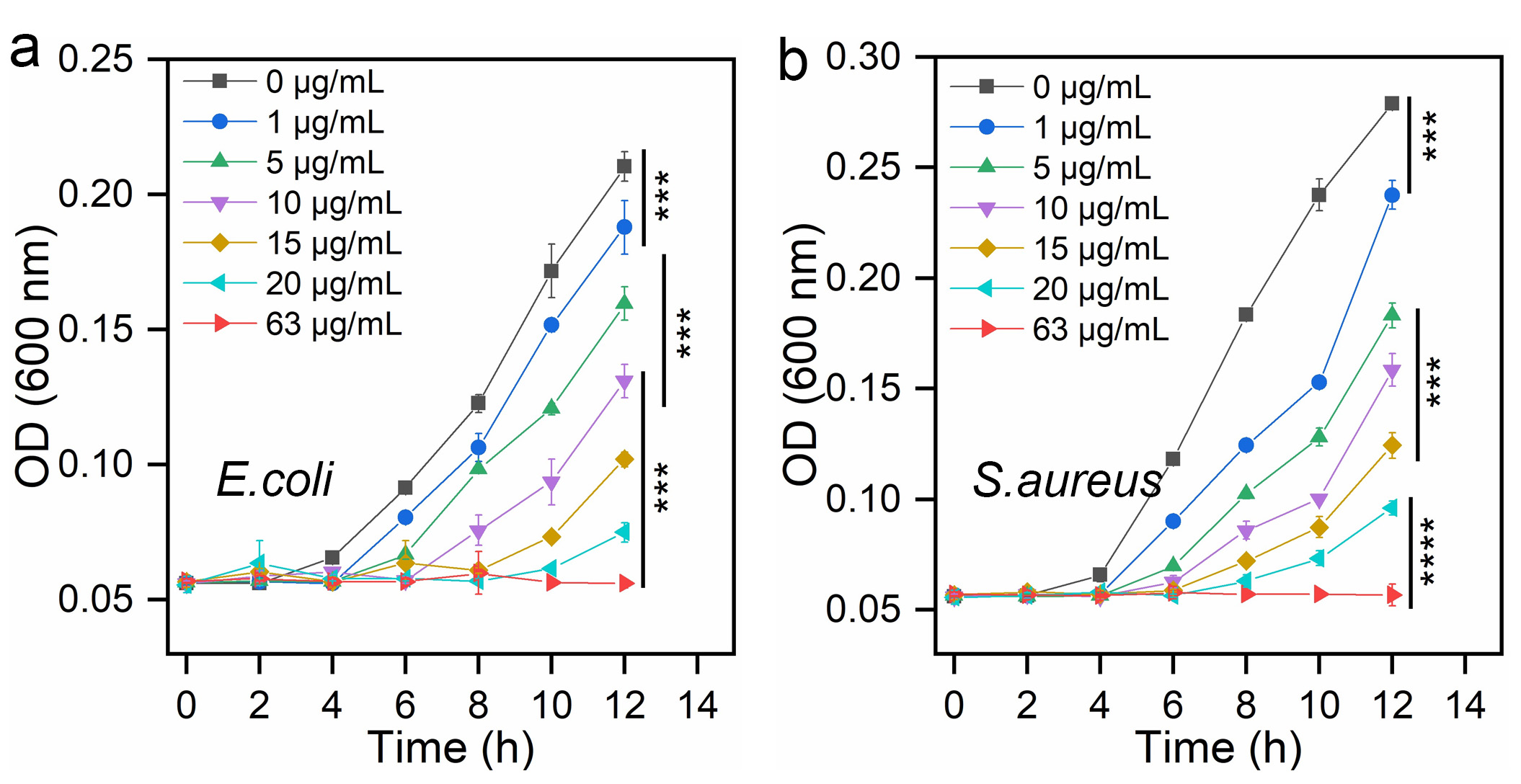


**Fig. S14.** **The growth curves of (a) *E. coli* and (b) *S. aureus* after incubation with different concentrations (from 0 to 63 μg mL^-1^) of Cuf-TMB@PDA.** It was demonstrated that a concentration of 63 μg mL^-1^ exhibited the most potent bacterial inhibitory effect (**P* < 0.05, ***P* < 0.01, ****P* < 0.001).





**Fig. S15. Evaluation of the antimicrobial activities of TiO_2_, Ag, vancomycin antibiotic, Cuf-TMB, and Cuf-TMB@PDA.** The antimicrobial property of Cuf-TMB and Cuf-TMB@PDA is comparable to that of the antimicrobial agent vancomycin (**P < 0.05, **P < 0.01, ***P < 0.001*).





**Fig. S16. The ESR spectra of Cuf-TMB@PDA and Cuf-TMB.** The signal intensity of free radicals was measured using 5,5-dimethyl-1-pyrroline-n-oxide (DMPO) as the spin-trap. It was observed that the Cuf-TMB system contained substantial quantities of ·OH and ·CHO radicals, and a portion of these ·OH radicals were still detectable in the Cuf-TMB@PDA system. This phenomenon could be attributed to the potential consumption of ·OH and ·CHO radicals during the formation of Cuf-TMB@PDA.


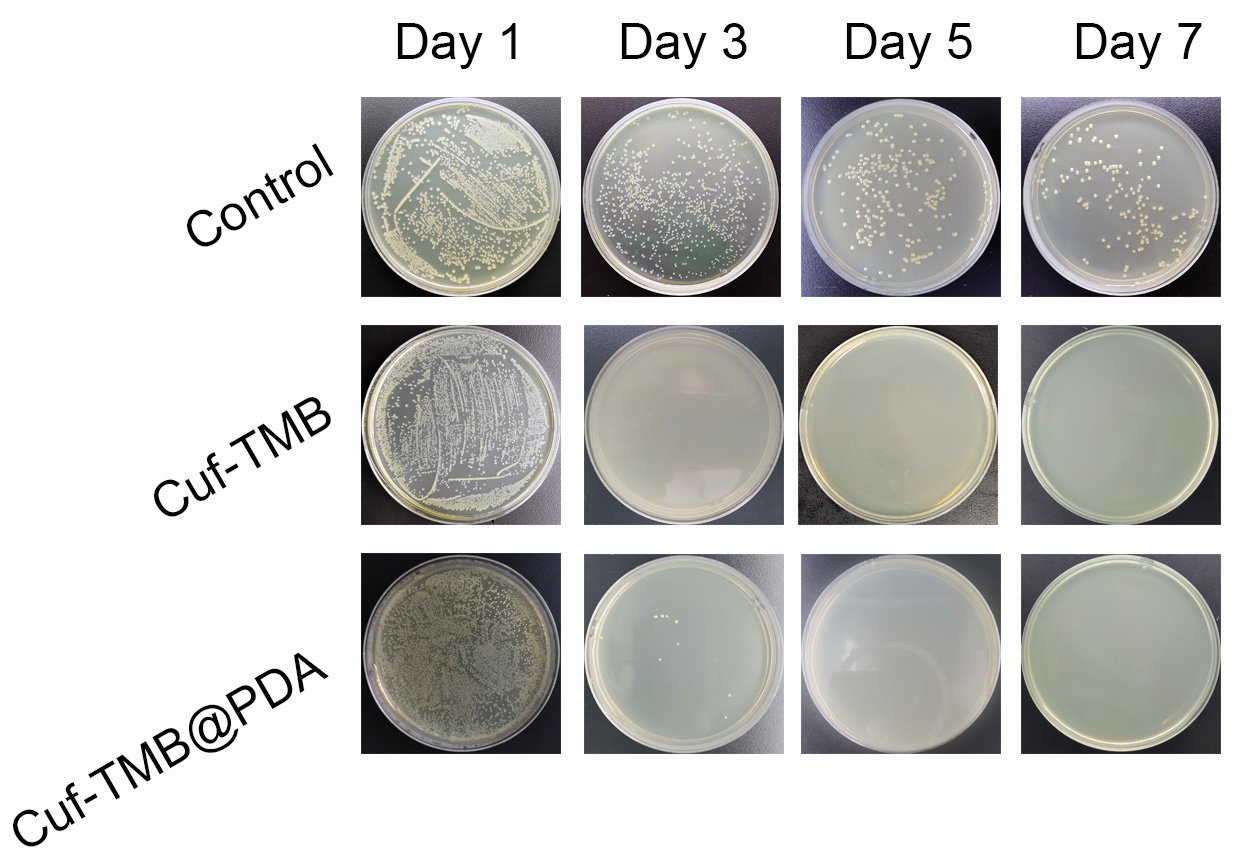


**Fig. S17. Representative photographs of bacterial cultures taken from *S. aureus* infected wound areas at different intervals during the treatment process.** The presence of bacteria at the wound sites was assessed using the agar plate method to measure the bactericidal effect. In both the Cuf-TMB and Cuf-TMB@PDA treatment groups, a significant reduction in the number of colonies collected from mouse wounds was observed. This effectively prevented wound infections and facilitated the wound healing process.





**Fig. S18. Body weight changes of *S. aureus* infected mice in various groups over 7 days.**

**Reference**

1. Qi C, Zhang Y, Tu J: Facile synthesis of ε-poly-^L^-lysine-conjugated ZnO@PDA as photothermal antibacterial agents for synergistic bacteria killing and biofilm eradication. *Biochem. Eng. J.* 2022; 186:108569.
2. Jiang X, Li S, Xiang G, Li Q, Fan L, He L, Gu K: Determination of the acid values of edible oils via FTIR spectroscopy based on the OH stretching band. *Food Chem.* 2016; 212:585-589.
3. Geng X, Xie X, Liang Y, Li Z, Yang K, Tao J, Zhang H, Wang Z: Facile fabrication of a novel copper nanozyme for efficient dye degradation. *ACS Omega.* 2021; 6(9):6284-6291.
4. Varoujan A. Y., Ashraf A. I.: Investigation of the enolization and carbonyl group migration in reducing sugars by FTIR spectroscopy. *Carbohyd. Res.* 1995; 276:253-265.
5. V. Guichard, A. Bourkba, O. Poizat: Vibrational Studies of Reactive Intermediates of Aromatic Amines. 2. Free-Radical Cation and Dlcation Resonance Raman Spectroscopy of *N, N, N', N'*-Tetramethylbenzidine and *N, N, N', N'*-Tetraethylbenzidine. *J. Phys. Chem.* 1989; 93:4429-4435.
6. Tian B, Zhao L, Li R, Zhai T, Zhang N, Duan Z, Tan L: Electrochemical immunoassay of endothelin-1 based on a fenton-type reaction using cu(ii)-containing nanocomposites as nanozymes. *Anal Chem.* 2020; 92(24):15916-15926.
7. Jiang Q, Xiong P, Liu J, Xie Z, Wang Q, Yang XQ, Hu E, Cao Y, Sun J, Xu Y et al: A redox-active 2d metal-organic framework for efficient lithium storage with extraordinary high capacity. *Angew. Chem. Int. Ed.* 2020; 59(13):5273-5277.
